# Structure–Activity Relationship Studies in a Series of Xanthine Inhibitors of SLACK Potassium Channels

**DOI:** 10.3390/molecules29112437

**Published:** 2024-05-22

**Authors:** Alshaima’a M. Qunies, Brittany D. Spitznagel, Yu Du, Paul K. Peprah, Yasmeen K. Mohamed, C. David Weaver, Kyle A. Emmitte

**Affiliations:** 1 Department of Pharmaceutical Sciences, UNT System College of Pharmacy, University of North Texas Health Science Center, Fort Worth, TX 76107, USA; 2 School of Biomedical Sciences, University of North Texas Health Science Center, Fort Worth, TX 76107, USA; 3 Department of Pharmacology, Vanderbilt University, Nashville, TN 37232, USA; 4 Vanderbilt Institute for Chemical Biology, Vanderbilt University, Nashville, TN 37232, USA

**Keywords:** SLACK, *KCNT1*, K_Na_1.1, Slo2.2, CNS, EIMFS, MMPSI, xanthine, epilepsy, neuroscience

## Abstract

Gain-of-function mutations in the *KCNT1* gene, which encodes the sodium-activated potassium channel known as SLACK, are associated with the rare but devastating developmental and epileptic encephalopathy known as epilepsy of infancy with migrating focal seizures (EIMFS). The design of small molecule inhibitors of SLACK channels represents a potential therapeutic approach to the treatment of EIMFS, other childhood epilepsies, and developmental disorders. Herein, we describe a hit optimization effort centered on a xanthine SLACK inhibitor (**8**) discovered via a high-throughput screen. Across three distinct regions of the chemotype, we synthesized 58 new analogs and tested each one in a whole-cell automated patch-clamp assay to develop structure–activity relationships for inhibition of SLACK channels. We further evaluated selected analogs for their selectivity versus a variety of other ion channels and for their activity versus clinically relevant SLACK mutants. Selectivity within the series was quite good, including versus hERG. Analog **80** (VU0948578) was a potent inhibitor of WT, A934T, and G288S SLACK, with IC_50_ values between 0.59 and 0.71 µM across these variants. VU0948578 represents a useful in vitro tool compound from a chemotype that is distinct from previously reported small molecule inhibitors of SLACK channels.

## 1. Introduction

Epilepsy of infancy with migrating focal seizures (EIMFS), previously known as malignant migrating partial seizures of infancy (MMPSI), is a catastrophic and rare epileptic disorder affecting infants and children [1]. Patients diagnosed with EIFMS commonly experience seizures that are resistant to standard anti-seizure medications within the first six months of life [2,3,4]. These seizures are persistent, originate in multiple focal areas, and shift across various regions within the brain. The progression of EIFMS is often marked by a decline in motor and cognitive development, visual disturbances, and muscle weakness. The etiology of EIMFS has been linked to a series of de novo missense gain-of-function (GOF) mutations in the *KCNT1* gene in roughly half of patients. *KCNT1* encodes for a sodium (Na^+^)-activated potassium (K^+^) channel known as SLACK (Sequence Like A Calcium-activated K^+^ channel), which is a critical regulator of electrical conductance within the central nervous system (CNS). SLACK plays a pivotal role in managing neural excitability, including the process of afterhyperpolarization (AHP) following recurrent neural discharges, which is critical for the modulation of action potential frequencies [2,3,4,5,6,7].

SLACK (K_Na_1.1, Slo2.2) belongs to the Slo family of K^+^ channels, which also includes Slo1 (Maxi-K, BK, or K_Ca_1.1), Slo2.1 (SLICK), and Slo3 [6,8,9]. SLACK channels are widely distributed within the CNS and are also present in certain peripheral tissues such as the gonads, muscle cells, cardiac myocytes, and the pituitary glands [2,6,8]. Structurally, SLACK channels are composed of a tetrameric assembly of subunits, each featuring six hydrophobic transmembrane segments (S1 to S6) and a pore-forming domain between S5 and S6. Also present is an extensive C-terminal cytoplasmic domain, which includes two tandem regulator of potassium conductance (RCK) domains that impart Na^+^ sensitivity to the channels, alongside a nicotinamide adenine dinucleotide (NAD^+^) binding domain [3,5,9,10,11,12,13,14]. As expected, intracellular concentration of Na^+^ is a key determinant of the activity of SLACK channels [12,14]; nevertheless, their activity is further modulated by the voltage across the membrane, direct phosphorylation by protein kinase C (PKC), indirect modulation by protein kinase A (PKA), and varying levels of cytoplasmic NAD^+^, ATP, and estradiol, among others [12,15,16]. 

More than thirty distinct *KCNT1* GOF mutations have been identified, all of which are linked to EIMFS [2,17,18,19,20]. These mutations predominantly occur within the C-terminal domain of the protein, as well as within the transmembrane pore domain [2,7,9,13,21,22]. The cytoplasmic domain is known to interact with developmental proteins, including the fragile X mental retardation protein (FMRP), the cytoplasmic FMR1-interacting protein 1 (Cyfip1), and the actin regulator 1 (Phactr1) [23,24]. Moreover, GOF mutations in *KCNT1* have been linked to a spectrum of epileptic disorders beyond EIMFS, such as early onset epileptic encephalopathy (including West syndrome and Ohtahara syndrome) and autosomal dominant nocturnal frontal lobe epilepsy [22,25,26,27,28]. Multiple hypotheses have been put forth regarding the mechanisms by which GOF mutations in the *KCNT1* gene lead to increased excitability of neurons. For example, these mutations may contribute to impaired function of inhibitory interneurons, thereby reducing the activity of these critical regulatory neurons. Likewise, GOF mutations have been shown to cause increased amplitude of AHP, which in turn increases the frequency of neuronal firing. Lastly, certain mutations may also interfere with cell signaling pathways that involve proteins such as FMRP and Phactr-1 [13,17,20,29].

**Figure 1:**
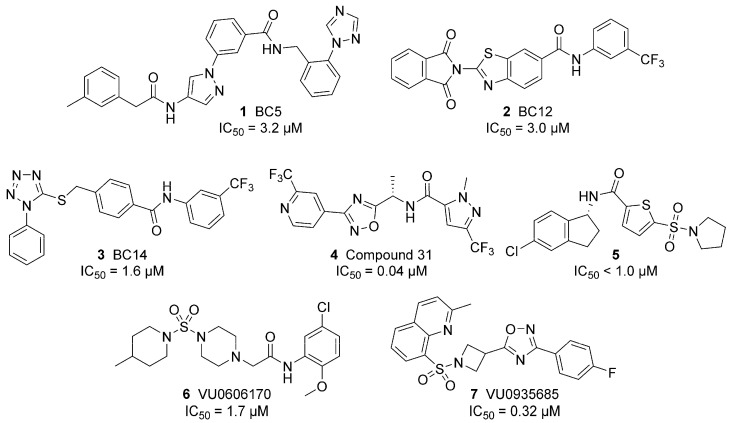
Examples of small molecule SLACK inhibitors reported in the literature. Reported IC_50_ values appear determined using whole-cell patch-clamp electrophysiology versus WT SLACK. Refer to original references cited in the text for experimental details as cell lines and protocols differ. Note the prevalence of secondary amides and five-membered heteroaryl rings across chemotypes.

To date, effective and safe therapeutic options remain unavailable for the treatment of EIMFS. While older, non-selective medications such as quinidine, clofilium, and bepridil inhibit SLACK currents in vitro, they have limited efficacy in the clinic, and quinidine causes toxicity due to its lack of selectivity and unfavorable pharmacokinetic properties [4,7,13,18,28,30,31,32,33,34,35,36]. Not surprisingly, multiple research groups have endeavored to identify new small molecules that may serve as lead compounds for the discovery of SLACK inhibitors. Recently, such efforts have begun to bear fruit. For example, researchers from the University of Leeds have identified potential SLACK pore blockers utilizing a virtual screen of 100,000 compounds via a cryo-electron microscopy-derived chicken SLACK structure. Six chemically diverse compounds with micromolar inhibitory potency in whole-cell patch-clamp assays were identified (compounds **1**–**3** in Figure 1; the remaining three compounds were noted as pan-assay interference compounds (PAINS) [37,38,39]). Compound **1** (BC5 in the original publication) almost completely inhibited hERG (K_V_11.1) channels at 10 µM while inhibition by **2** (BC12 in the original publication) and **3** (BC14 in the original publication) at the same concentration was more moderate, at 10–20% and 45%, respectively [40].

Praxis Precision Medicine has been actively working toward the design of small molecule SLACK inhibitors for several years and has recently published in the peer-reviewed [41] as well as patent literature [42]. 1,2,4-Oxadiazole-based in vivo probe **4** (Figure 1, Compound **31** in the original publication) was evaluated via whole-cell automated patch-clamp (APC) versus a variety of human and murine WT and SLACK GOF variants and showed IC_50_ values between 0.040 µM and 1.77 µM. However, compound **4** also exhibited moderate potency as an inhibitor of the hERG channel with an IC_50_ value of 11.9 µM. Importantly, compound **4** demonstrated anticonvulsant effects in a mouse model using *Kcnt1*^L/L^ mice [43]. Of note, **4** also exhibited 74% displacement at GABA_A_ Cl^–^ channels in a binding displacement assay, hinting at a potential contribution from GABAergic modulation in mediating its anticonvulsant effects [41]. 2,3-Dihydro-1*H*-indene **5** (Figure 1**,** Compound I-17 in the published patent application) is representative of a distinct series of SLACK inhibitors from Praxis that was disclosed in the patent literature and reported to have an IC_50_ value less than one micromolar in the APC assay [44].

We inaugurated our efforts to discover novel inhibitors of SLACK channels with a high-throughput screen (HTS) of approximately 100,000 compounds using a fluorescence-based thallium (Tl^+^) flux assay [45] with HEK-293 cells that stably express WT SLACK. Among the hits identified was 2-amino-*N*-phenylacetamide inhibitor **6** (Figure 1, VU0606170) [9]. Despite extensive optimization efforts within this scaffold, we failed to identify analogs notably superior to the hit compound. Nonetheless, VU0606170 and several related analogs exhibited micromolar potency in a whole-cell APC assay using the same WT SLACK-expressing HEK-293 cells. This same set of analogs demonstrated activity in a Tl^+^-flux assay utilizing A934T SLACK-expressing HEK-293 cells with equivalent or superior potency as observed versus WT SLACK [15]. Finally, in a phenotypic assay of epilepsy [46] employing co-cultures of cortical neuronal and glial cells, **6** inhibited the frequency of spontaneous calcium oscillations, which is considered an anti-epileptic phenotype [9]. Compound **6** inhibited the hERG channel in a Tl^+^-flux assay with an IC_50_ value greater than 10 µM [9]. Recently, we also published substantial hit optimization work in our own 1,2,4-oxadiazole series of SLACK inhibitors that culminated in the identification of optimized in vitro tool **7** (Figure 1, VU0935685) [47]. Tool compound **7** demonstrated improved potency (IC_50_ = 0.32 µM) in a whole-cell APC assay using CHO cells stably expressing WT SLACK. Evaluation of **7** in Tl^+^-flux assays likewise showed excellent potency versus A934T SLACK (IC_50_ = 0.67 µM) with good selectivity versus Slo family members SLICK and Maxi-K as well as hERG. Still, **7** was rapidly metabolized in mouse liver microsomes, pointing to the need for continued optimization [47].

## 2. Results and Discussion

### 2.1. Selection of Hit Compound VU0607689

**Figure 2:**
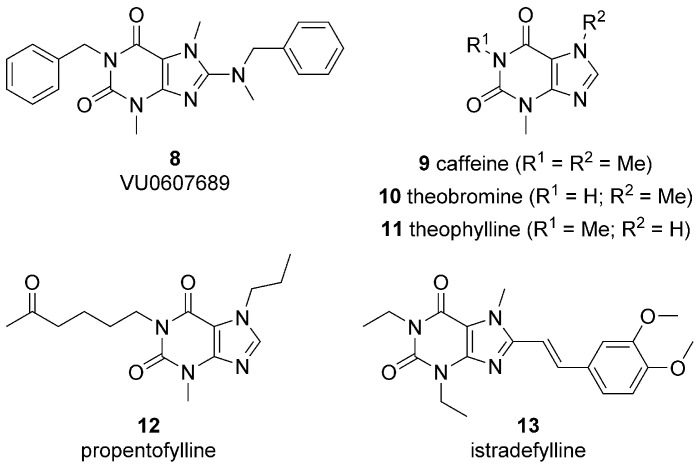
HTS SLACK inhibitor hit **8** (VU0607689); simple xanthine compounds caffeine (**9**), theobromine (**10**), and theophylline (**11**); propentofylline (**12**), a xanthine inhibitor of equilibrative nucleoside transporters used in veterinary medicine; and istradefylline (**13**), a xanthine inhibitor of adenosine A_2A_ receptors used in the treatment of Parkinson’s disease.

Having access to multiple high-quality small molecule probes from distinct chemotypes is invaluable in the preclinical validation of a novel target. Thus, we were eager to continue our efforts toward the discovery of novel small molecule inhibitors of SLACK channels. We quickly became interested in xanthine hit **8** (Figure 2, VU0607689) which was also discovered during our HTS campaign. While it is well known that simple xanthines such as caffeine, theobromine, and theophylline (Figure 2, **9**–**11**) readily cross the blood–brain barrier (BBB) [48], it has also been established that more elaborated and functionalized xanthine-based molecules, such as propentofylline and istradefylline (Figure 2, **12** and **13**), can be developed as ligands that engage specific receptors in the CNS via passive permeability of the BBB [49,50,51]. In fact, hit compound **8** has several calculated properties (obtained via PubChem, CID: 16812252) consistent with good permeability. For example, molecular weight (389.4 Da), cLogP (3.0), H-bond donors (0), and H-bond acceptors (4) are all within Lipinski’s guidelines [52]. Likewise, the topological polar surface area (61.7 Å^2^) and number of rotatable bonds (5) of **8** are predictive of good oral absorption and BBB permeability [53,54]. Calculation of the recently described BBB score [55] gave a value of 4.6, which, being between 4 and 6, is predictive of CNS penetrance. Finally, follow-up screening of a resynthesized batch of **8** in our APC assay showed good potency versus WT SLACK (IC_50_ = 1.7 µM). 

**Figure 3:**
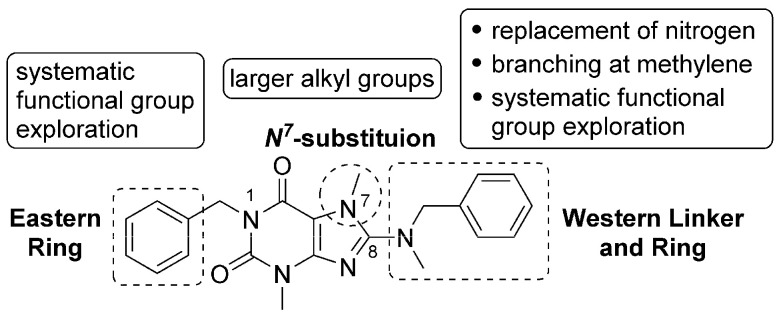
Hit optimization plan for SLACK inhibitor VU0607689 in three regions.

Buoyed by the potential of the scaffold, we set out to develop SAR within this chemotype via a parallel hit optimization strategy centered on three regions (Figure 3). Without knowledge of the binding site of the hit compound, strategies such as molecular docking were viewed as unlikely to prove fruitful. Thus, our strategy was to be thorough in our investigation of each region and to remain agnostic as to which functional groups may or may not be preferred. Attached to the N^1^ atom of the core is a benzyl moiety we termed the eastern ring of the scaffold. Here, we planned to systematically investigate substitution at each of the positions with a variety of functional groups of varying steric and electronic character. Within the core of the scaffold, we planned to evaluate the impact of larger alkyl groups at the N^7^ atom. Finally, in the western linker and ring system attached to the C^8^-position of the core, we planned to explore multiple modifications. For example in the linker region, we were interested in replacing the nitrogen atom with carbon and oxygen as well as exploring alkyl branching at the methylene group. Likewise, we also planned systematic exploration of the terminal phenyl group in a similar manner to that outlined for the eastern ring.

### 2.2. Synthesis of Analogs

**Scheme 1:**
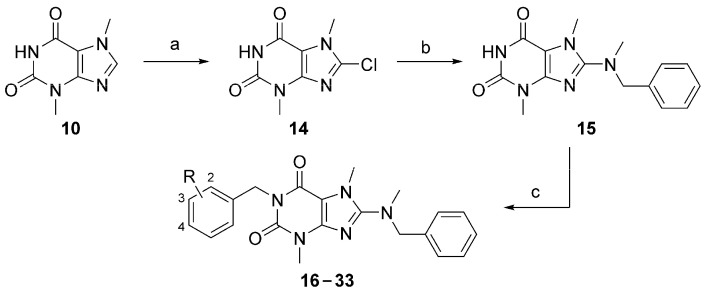
Synthesis of eastern ring analogs. *Reagents and Conditions:* (a) NCS, THF, 60 °C, *74%*; (b) *N*-methylbenzylamine, Cs_2_CO_3_, DMF, microwave, 130 °C, 30 min, *43%*; (c) (2-F-Ph)CH_2_OMs, NaH, THF, *36%* (**16**); (3-F-Ph)CH_2_OTs, NaH, DMF, *22%* (**17**); ArCH_2_Cl, Cs_2_CO_3_, DMF, *4–48%* (**18**–**20**, **22**–**27**, and **31**–**33**); ArCH_2_Br, K_2_CO_3_, DMF, *9–71%* (**21** and **28**–**30**).

Synthesis of eastern ring analogs is depicted in Scheme 1 and began with commercially available theobromine (**10**), which was chlorinated at C^8^-position with *N*-chlorosuccinimide to afford intermediate **14** in good yield. Reaction of **14** with *N*-methylbenzylamine in the presence of cesium carbonate utilizing microwave-assisted organic synthesis (MAOS) [56] facilitated the nucleophilic aromatic substitution (S_N_Ar) to provide penultimate intermediate **15** in moderate yield. Alkylation of **15** was accomplished under basic conditions via reaction with the appropriately substituted benzyl mesylate, benzyl tosylate, benzyl chloride, or benzyl bromide to yield the target analogs **16**–**33**. 

**Scheme 2:**
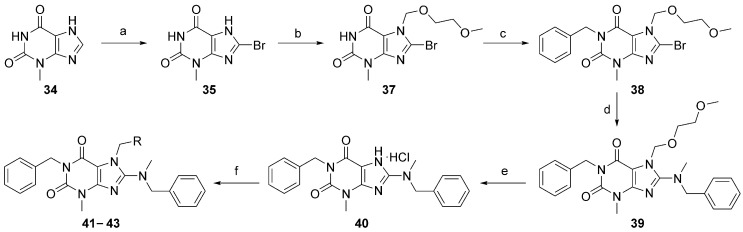
Synthesis of *N*^7^-position analogs. *Reagents and Conditions:* (a) Br_2_, AcOH, 100 °C, *95%*; (b) ClCH_2_OCH_2_CH_2_OCH_3_ (**36**), NEt_3_, DMF (c) BnBr, K_2_CO_3_, 100 °C, *34%* over 2 steps; (d) *N*-methylbenzyl amine, Cs_2_CO_3_, DMF, 100 °C, *85%*; (e) *4N* HCl, 1,4-dioxane, *93%*; (f) EtI, Cs_2_CO_3_, DMF, microwave, 130 °C, 30 min, *9%* (**41**); RCH_2_Br, NaH, DMF, *31%* (**42**), *28%* (**43**).

Synthesis of analogs at the N^7^-position of the core was carried out as shown in Scheme 2. Bromination of 3-methylxanthine **34** proceeded smoothly to provide intermediate **35**. The nitrogen atom at the 7-position of the core was then protected with MEM chloride (**36**) to afford **37**. In the same pot, alkylation of the 1-position nitrogen atom was accomplished with benzyl bromide to give **38** in moderate yield over two steps. Reaction of **38** with *N*-methylbenzylamine in the presence of cesium carbonate with thermal heating efficiently effected the S_N_Ar reaction, affording **39** in high yield. Cleavage of the MEM protecting was accomplished under acidic conditions to give penultimate intermediate **40** as its hydrochloride salt. Conversion of **40** into analogs **41**–**43** was accomplished via alkylation with the appropriate alkyl halide.

**Scheme 3:**
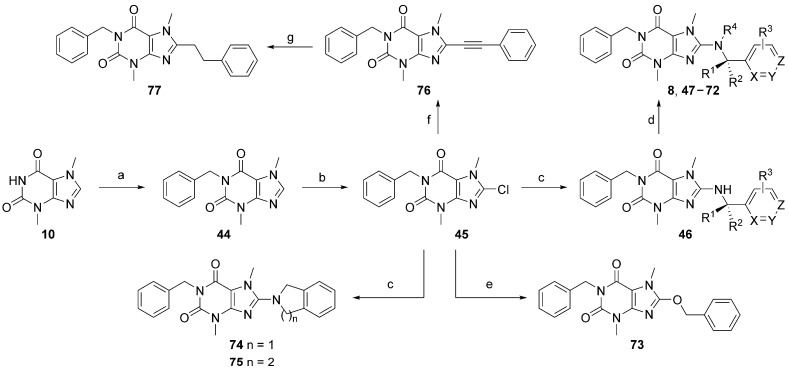
Synthesis of western linker and ring analogs. *Reagents and Conditions:* (a) BnBr K_2_CO_3_, DMF, 90 °C, *95%*; (b) NCS, THF, 60 °C, *71%*; (c) 1° or 2° benzylamine, Cs_2_CO_3_, DMF, microwave, 130 °C, 30 min, *34%* (**74**), *32%* (**75**) (d) MeI, NaH, THF, *4–37%* over 2 steps (**8**, **47**–**66**); R^4^X (X = Br or I), NaH, THF, 0 to 50 °C, *8–40%* (**67**–**72**); (e) PhCH_2_OH, Cs_2_CO_3_, DMF, microwave, 130 °C, 30 min, *64%*; (f) PhC≡CH, Pd(PPh_3_)_4_, NEt_3_, CuI, DMF, 100 °C, *47%*; (g) 10% Pd/C, MeOH, 10 bar, 60 °C, H-Cube^®^, *23%*.

Synthesis of analogs in the western linker and ring of the scaffold followed the methodology outlined in Scheme 3. Once again, we began with commercial theobromine **10**; however, in this case, the sequence began with benzylation of the N^1^ atom, which proceeded in excellent yield to afford **44**. Chlorination at the C^8^ position gave key intermediate **45** in good yield. From compound **45**, we were able to access a variety of different analogs. For example, S_N_Ar reaction of **45** with a host of commercially available primary benzyl amines was carried out using MAOS [56] to provide intermediates **46**. In the case of monomers containing substituents (R^3^) on the phenyl ring or pyridine replacements for the phenyl ring (X, Y, or Z = N), reaction of **46** with methyl iodide gave analogs **47**–**64** in low to moderate yield. Likewise, we also carried out the same two-step sequence with both enantiomers of 1-phenethylamine to access chiral analogs **65** and **66**. To evaluate SAR at the linker nitrogen (R^4^), we followed the addition of benzylamine by reaction with a variety of alkylating agents to provide analogs **67**–**72**. Finally, the S_N_Ar reaction of **45** with isoindoline and 1,2,3,4 tetrahydroisoquinoline provided analogs **74** and **75**, respectively, in moderate yield. Oxygen linker analog **73** was prepared in good yield through reaction of **45** with benzyl alcohol using MAOS [56]. To access the carbon linker analogs, a Sonogashira coupling [57] between ethynyl benzene and **45** was used to prepare **76** in moderate yield. Exhaustive hydrogenation of **76** under palladium catalysis gave analog **77** in low yield.

### 2.3. Structure–Activity Relationships

All new analogs were tested in our APC assay using CHO cells stably expressing WT SLACK using a concentration-response format with concentrations ranging from 0.125 to 30 μM in half-log steps and a minimum of three cells (replicates) per concentration series. In the eastern ring, the systematic substitution of the ring showed the SAR to be relatively flat with many analogs being within two-fold of the activity observed with the hit compound **8** (Table 1). Still, there were some notable results. For example, substitution at the 4-position was largely unfavorable regardless of the nature of the substituent (**21**, **24**, **27**, **30**, and **33**) apart from the 4-fluoro analog **18**. Interestingly, substitution with the trifluoromethyl group (**25**–**27**) was unfavorable at all positions, especially at the 2- and 4-positions, and inferior to the corresponding chloro (**19**–**21**) and methyl analogs (**22**–**24**). As the trifluoromethyl group is similar in electronegativity to chlorine, but larger than both methyl and chlorine [58,59], perhaps the steric bulk of the trifluoromethyl is to blame for the poor potency of these analogs. With the exception of the fluoro (**16**–**18**) and cyano (**28**–**30**) analogs, substitution at the 3-position was favorable for SLACK activity, with 3-methoxy analog **32** standing out in this set. Alkylation of the N^7^-position of the core with any groups larger than methyl proved universally detrimental to SLACK activity (Table 2). 

Systematic functionalization of the western ring of the scaffold revealed few analogs with improved SLACK activity relative to hit compound **8** (Table 3). Installation of a heteroatom in the ring at any position (**62**–**64**) proved particularly intolerable. In stark contrast to the results obtained in the eastern ring (**25**–**27**, Table 1), trifluoromethyl analogs (**56**–**58**) were well tolerated here. In fact, outside of the 2-trifluoromethyl analog **56**, substitution at the 2-position (**47**, **50**, **53**, and **59**) reduced potency at SLACK, with the most notable reductions in activity observed with electron-donating methyl (**53**) and methoxy (**59**) groups. In most cases, substitution at the 3- and 4-positions was tolerated regardless of the functional group, though 4-chloro (**52**) and 3- and 4-methoxy (**60** and **61**) substitution was moderately disfavored. Among this set, only 3-fluoro analog **48** stood out as markedly improved compared to the hit compound **8**.

Evaluation of structural changes to the western linker provided quite divergent SAR with respect to SLACK potency, both favorable and unfavorable (Table 4). Testing of secondary amine intermediate **46a** demonstrated the necessity of a tertiary amine at the C^8^-position for SLACK activity. Of particular note were the results observed with chiral analogs **65** and **66**. Although (*R*)-enantiomer **65** was equipotent to hit **8**, it was more than six-fold more potent than (*S*)-enantiomer **66**. Perhaps this instance of stereochemical bias would be worthy of future follow-up to explore whether other alkyl groups in the (*R*)-configuration yielded improvements in potency. While branching (**69**) of the alkyl group at the carbon immediately adjacent to the nitrogen atom was not favorable, other alkyl groups larger than methyl (**67**, **68**, **70**–**72**) improved SLACK activity, with *n*-butyl (**70**) and cyclopropylmethyl (**71**) analogs standing out within this set. Replacement of the nitrogen atom with oxygen (**73**) and carbon (**76** and **77**) was not favorable for SLACK activity. Interestingly, while isoindolin-2-yl analog **74** was inactive, expansion of the saturated ring from a five- to six-membered ring in analog **75** restored most of the lost potency. 

### 2.4. Secondary Pharmacological Profiling

With a handful of analogs that offered improved SLACK potency relative to hit compound **8**, we next proceeded to investigate the ancillary pharmacology of such compounds, both with respect to other Slo family members as well as versus a spectrum of diverse ion channels (Table 5). Thus, we screened four compounds (**32**, **48**, **70**, and **71**) at 10 µM in our in-house selectivity panel of Tl^+^-flux assays in HEK-293 cells stably expressing the target of interest [9,60]. The selectivity profile for all four analogs was largely encouraging, with few instances of greater than 50% inhibition observed at 10 µM. Some valuable SAR was noted as well. For example, while *N*-methyl analogs **32** and **48** demonstrated notable inhibition of Maxi-K α1/β3 at 79% and 63%, respectively, the installation of the larger *n*-butyl (**70**) and methylcyclopropyl (**71**) groups on the nitrogen atom significantly reduced activity versus this target. Reduction of inhibition at Maxi-K α1/β4 also followed. This SAR will be important to bear in mind for continued optimization within this series. A similar SAR was observed with regard to inhibition of K_V_2.1 [61], another potassium channel with mutations linked to infantile epilepsy [62]. Also, within the Slo family, none of the four analogs demonstrated notable activity against SLICK even though it is highly homologous to SLACK, with 78% sequence identity [10]. Of particular note is the lack of significant hERG activity in this series, as activity versus that counter-target has been an issue with other SLACK inhibitors [9,40,41]. Thus, this xanthine series provides another example [47] within a distinct chemotype that demonstrates an ability to design potent SLACK inhibitors with selectivity versus the hERG channel. 

Development of a useful SLACK inhibitor in vivo tool will require potent activity at the corresponding mouse ortholog as studies in appropriate mouse models [20,63,64,65] will be critical. Thus, we evaluated analogs **32** and **48** in our APC assay using CHO cells stably expressing mouse WT SLACK (Table 6). Activity versus the mouse proved only marginally reduced compared to the human with both compounds. We were also eager to evaluate the same compounds with regard to their ability to inhibit clinically relevant GOF SLACK mutants. Both the C-terminus A934T mutant and transmembrane G288S mutant have been associated with clinical cases of EIMFS [3,7]. Gratifyingly, both **32** and **48** inhibited these mutant targets with similar potency as observed versus WT SLACK using our APC assay (Table 6).

### 2.5. Synthesis and Testing of Combination Analogs

Finally, we turned our attention to the synthesis of second-generation analogs that combined optimal features. In the eastern ring, we chose the 3-methoxy benzyl group found in analog **32**. At the nitrogen atom appended to the C^8^-position of the core, we chose the *n*-butyl and methylcyclopropyl groups of analogs **70** and **71**, respectively. In the western ring, we chose the 3-fluoro and 4-fluoro groups of analogs **48** and **49**, respectively. Using chemistry analogous to that described in Scheme 3, we prepared analogs **78**–**81** (Table 7). Evaluation of these four analogs using APC versus WT, A934T, and G288S revealed that the combination of structural changes in the scaffold led to improved activities in some cases. Most notably, *n*-butyl analogs **78** and **80** both showed improved potency versus the A934T mutant compared to **32** and **48**. 

### 2.6. Conclusions and Future Directions

In conclusion, we have executed a hit optimization strategy centered on xanthine HTS hit **8** and identified substitution of the eastern and western rings as well as alkylation of the nitrogen atom group at the C^8^-position as viable strategies for increasing inhibitory potency of SLACK channels. Chemistry employed to access key intermediates involved moderate to high yielding reactions, facilitating the synthesis of analogs. Isolated yields for the conversion of penultimate intermediates into final compounds varied more widely and were poor in some cases. In certain instances, poor yields could be attributed to low conversion or impurities that complicated the isolation of pure samples. Selectivity profiling versus Slo family channels and a diverse set of other ion channels was encouraging, with SAR identified for reducing activity at Maxi-K and K_V_2.1. Selectivity in this series versus hERG was also promising, providing another scaffold that demonstrates potential in this key area. Finally, synthesis of analogs that combined optimized features in different regions yielded molecules with enhanced potency versus the clinically relevant A934T SLACK mutant. In fact, analog **80** demonstrated inhibitory potency between 0.59 and 0.71 µM versus WT, A934T, and G288S SLACK. Thus, compound **80** (VU0948578) represents a valuable in vitro tool from a series wholly distinct from SLACK inhibitors disclosed to date. The next step for this series must include evaluation of the metabolic and physicochemical properties of representative compounds within the series to evaluate any potential liabilities in areas such as clearance, solubility, and permeability. The results from such studies will enable an assessment of the probability that continued optimization efforts in the series may lead to the discovery of a molecule suitable for use as an in vivo probe.

## 3. Materials and Methods

### 3.1. Synthesis and Purification

Air-sensitive reactions were carried out under a nitrogen atmosphere. Starting materials, reagents, intermediates, and final compounds were weighed on a Mettler Toledo^TM^ New Classic ME analytical balance or a Mettler Toledo^TM^ New Classic ME toploader balance. Thin-layer chromatography (TLC) was conducted on glass plates coated with Silica Gel 60 F_254_ from Millipore Sigma. Normal-phase flash chromatography was carried out on either a CombiFlash^®^ EZ Prep or CombiFlash^®^ Rf+ automated flash chromatography system, both from Teledyne ISCO. Normal-phase flash chromatography was carried out using Redi*Sep*^®^ Rf normal-phase, disposable flash columns from Teledyne ISCO or SiliaSep normal-phase, disposable flash columns (40–63 micron) from SiliCycle, Inc. Reverse-phase preparative chromatography was carried out on the CombiFlash^®^ EZ Prep using a reusable Redi*Sep*^®^ Rf C18 reverse-phase column. Microwave reactions were carried out an Anton Paar Monowave 200 automated microwave synthesizer. The Monowave 200 has an output power of 850 W with a maximum temperature of 260 °C and a maximum pressure of 290 psi and is suitable for use with reaction volumes ranging from 0.5 to 20 mL.

All NMR spectra were recorded on a 300 MHz Bruker Fourier 300HD NMR spectrometer equipped with a dual ^1^H and ^13^C probe with Z-Gradient and automatic tuning and matching, full computer control of all shims with TopShim^TM^, 24-sample SampleCase^TM^ automation system, and TopSpin^TM^ software, version 3.6.2. All NMR samples were prepared with either chloroform-d with 0.03% TMS (99.8+ atom % D, Acros Organics Catalog No. 209561000) or d_6_-dimethyl sulfoxide with 0.03% TMS (ACROS Organics Catalog No. 360000100). ^1^H and ^13^C chemical shifts are reported in δ values in ppm downfield. Data are reported as follows: chemical shift, multiplicity (s = singlet, d = doublet, t = triplet, q = quartet, br = broad, m = multiplet), integration, coupling constant (Hz). High resolution mass spectrometry was conducted on an Agilent 6230 Accurate-Mass Time-of-Flight (TOF) LC/MS with ESI source equipped with MassHunter Walkup software, version B.09.00, Build 9.0.9044.0. MS parameters were as follows: fragmentor: 175 V, capillary voltage: 3500 V, nebulizer pressure: 35 psig, drying gas flow: 11 L/min, drying gas temperature: 325 °C. Samples were introduced via an Agilent 1260 Infinity UHPLC comprised of a G4225A HiP Degasser, G1312B binary pump, G1367E ALS, G1316A TCC, and G1315C DAD VL+ with a 5 μL semi-micro flow cell with a 6 mm path length. UV absorption was observed at 220 nm and 254 nm with a 4 nm bandwidth. Column: Agilent Zorbax SB-C18, Rapid Resolution HT, 1.8 µm, 2.1 × 50 mm. Gradient conditions: hold at 5% CH_3_CN in H_2_O (0.1% formic acid) for 1.0 min, 5% to 95% CH_3_CN in H_2_O (0.1% formic acid) over 5 min, hold at 95% CH_3_CN in H_2_O (0.1% formic acid) for 1.0 min, 0.5 mL/min. All samples submitted for biological testing were confirmed ≥95% pure by ^1^H NMR.

### 3.2. Analog Synthesis 

Synthesis of analogs **16**–**18**, **21**, **8**, and **67** are described here as representative of the routes used to access the most active SLACK inhibitor compounds. Synthetic methods and characterization of all samples submitted for biological testing are available in the Supplementary Material. 

8-Chloro-3,7-dimethyl-3,7-dihydro-1H-purine-2,6-dione (**14**). Theobromine (**10**) (5.00 g, 27.8 mmol, 1.0 eq) and NCS (7.41 g, 55.5 mmol, 2.0 eq) were suspended in THF (25 mL), and the reaction was heated at 60 °C overnight. THF was removed in vacuo, and the residue was filtered and washed with water. The product was dried at 40 °C to provide 4.4 g (74%) of the title compound as a beige solid that was used further without purification. LCMS R_T_ = 2.62 min; HRMS, calc’d for C_7_H_8_ClN_4_O_2_^+^ [M + H], 215.0330; found 215.0330.

8-(Benzyl(methyl)amino)-3,7-dimethyl-3,7-dihydro-1H-purine-2,6-dione (**15**). Intermediate **14** (1.00 g, 4.66 mmol, 1.0 eq), N-methylbenzyl amine (1.13 g, 9.32 mmol, 2.0 eq), Cs_2_CO_3_ (3.03 g, 9.32 mmol, 2.0 eq), and DMF (5.0 mL) were added to a microwave vial and heated in a microwave reactor at 130 °C for 30 min. The reaction was cooled to room temperature, water was added, and the mixture was extracted with ethyl acetate (2×). The combined organics were washed with brine, dried over Na_2_SO_4_, filtered, and concentrated in vacuo. Purification of the residue by flash chromatography on silica gel yielded 600 mg (43%) of the title compound. LCMS R_T_ = 4.0 min; HRMS, calc’d for C_15_H_18_N_5_O_2_^+^ [M + H], 300.1455; found 300.1460.

8-(Benzyl(methyl)amino)-1-(2-fluorobenzyl)-3,7-dimethyl-3,7-dihydro-1H-purine-2,6-dione (**16**). Sodium hydride (8.0 mg, 0.34 mmol, 2.0 eq) was added to an ice-cold solution of **15** (50 mg, 0.17 mmol, 1.0 eq) and was stirred for 15 min. Afterwards, 2-fluorobenzyl methanesulfonate (69 mg, 0.34 mmol, 2.0 eq) was added, and the reaction was heated at 130 °C for one hour. The reaction was cooled to room temperature, water was added, and the mixture was extracted with DCM (2×). The combined organics were washed with brine, dried over Na_2_SO_4_, filtered, and concentrated in vacuo. Purification of the residue by flash chromatography on silica gel yielded 25 mg (36%) of the title compound. ^1^H NMR (300 MHz, CDCl_3_) δ 7.43–7.14 (m, 7H), 7.09–6.97 (m, 2H), 5.28 (s, 2H), 4.47 (s, 2H), 3.80 (s, 3H), 3.54 (s, 3H), 2.91 (s, 3H); ^13^C NMR (75 MHz, CDCl_3_) δ 162.33, 159.05, 157.58, 154.43, 151.61, 148.00, 136.77, 128.86 (d, *J*(C,F) = 4.07 Hz), 128.64 (d, *J*(C,F) = 8.29 Hz), 128.31 (d, *J*(C,F) = 69.62 Hz), 127.78, 124.68 (d, *J*(C,F) = 14.40 Hz), 124.00 (d, *J*(C,F) = 3.65 Hz), 115.35 (d, *J*(C,F) = 21.73 Hz), 105.03, 57.45, 38.91, 38.00 (d, *J*(C,F) = 4.97 Hz), 32.92, 29.76 ppm. LCMS R_T_ = 5.25 min; HRMS, calc’d for C_22_H_23_FN_5_O_2_^+^ [M + H], 408.1830; found 408.1836.

8-(Benzyl(methyl)amino)-1-(3-fluorobenzyl)-3,7-dimethyl-3,7-dihydro-1H-purine-2,6-dione (**17**). 3-Fluorobenzyl 4-methylbenzenesulfonate (95.3 mg, 0.34 mmol, 2.0 eq) was added to a solution of **15** (50 mg, 0.17 mmol, 1.0 eq) and Cs_2_CO_3_ (110 mg, 0.34 mmol, 2.0 eq) in THF (3 mL) and stirred at 60 °C overnight. The reaction was cooled to room temperature, water was added, and the mixture was extracted with DCM (2×). The combined organics were washed with brine, dried over Na_2_SO_4_, filtered, and concentrated in vacuo. Purification of the residue by flash chromatography on silica gel yielded 15 mg (22%) of the title compound. ^1^H NMR (300 MHz, CDCl_3_) δ 7.41–7.21 (m, 7H), 7.17 (m, 1H), 6.93 (m, 1H), 5.17 (s, 2H), 4.47 (s, 2H), 3.81 (s, 3H), 3.53 (s, 3H), 2.91 (s, 3H); ^13^C NMR (75 MHz, CDCl_3_) δ 162.77 (d, *J*(C,F) = 245.42 Hz), 157.60, 154.42, 151.63, 147.97, 140.25 (d, *J*(C,F) = 7.40 Hz), 136.75, 129.79 (d, *J*(C,F) = 8.25 Hz), 128.77, 127.84, 127.78, 124.21 (d, *J*(C,F) = 2.80 Hz), 115.41 (d, *J*(C,F) = 21.82 Hz), 114.23 (d, *J*(C,F) = 21.04 Hz), 105.05, 57.44, 42.74 (d, *J*(C,F) = 1.67 Hz), 38.91, 32.91, 29.74 ppm. LCMS R_T_ = 5.33 min; HRMS, calc’d for C_22_H_23_FN_5_O_2_^+^ [M + H], 408.1830; found 408.1835.

8-(Benzyl(methyl)amino)-1-(4-fluorobenzyl)-3,7-dimethyl-3,7-dihydro-1H-purine-2,6-dione (**18**). 4-Fluorobenzylchloride (49 mg, 0.34 mmol, 2.0 eq) was added to a solution of **15** (50 mg, 0.17 mmol, 1.0 eq) and Cs_2_CO_3_ (110 mg, 0.34 mmol, 2.0 eq) in DMF (0.5 mL) and stirred at 100 °C overnight. The reaction was cooled to room temperature, water was added, and the mixture was extracted with DCM (2×). The combined organics were washed with brine, dried over Na_2_SO_4_, filtered, and concentrated in vacuo. Purification of the residue by flash chromatography on silica gel yielded 28 mg (40%) of the title compound. ^1^H NMR (300 MHz, CDCl_3_) δ 7.49 (m, 2H), 7.41–7.27 (m, 5H), 6.97 (m, 2H), 5.14 (s, 2H), 4.46 (s, 2H), 3.80 (s, 3H), 3.52 (s, 3H), 2.90 (s, 3H); ^13^C NMR (75 MHz, CDCl_3_) δ 162.18 (d, *J*(C,F) = 245.07 Hz), 157.55, 154.50, 151.65, 147.88, 136.75, 133.65 (d, *J*(C,F) = 3.20 Hz), 130.74 (d, *J*(C,F) = 8.16 Hz), 128.76, 127.84, 127.77, 115.11 (d, *J*(C,F) = 21.19 Hz), 105.10, 57.45, 43.49, 38.92, 32.88, 29.71 ppm. LCMS R_T_ = 5.38 min; HRMS, calc’d for C_22_H_23_FN_5_O_2_^+^ [M + H], 408.1830; found 408.1830.

8-(Benzyl(methyl)amino)-1-(4-chlorobenzyl)-3,7-dimethyl-3,7-dihydro-1H-purine-2,6-dione (**21**). 4-Chlorobenzyl bromide (33 mg, 0.16 mmol, 2.0 eq) was added to a solution of **15** (25 mg, 0.08 mmol, 1.0 eq) and K_2_CO_3_ (22 mg, 0.16 mmol, 2.0 eq) in DMF (0.5 mL) and stirred at 100 °C overnight. The reaction was cooled to room temperature, water was added, and the mixture was extracted with DCM (2×). The combined organics were washed with brine, dried over sodium sulfate (Na_2_SO_4_), filtered, and concentrated in vacuo. Purification of the residue by flash chromatography on silica gel yielded 24 mg (71%) of the title compound. ^1^H NMR (300 MHz, CDCl_3_) δ 7.43 (m, 2H), 7.40–7.21 (m, 7H), 5.13 (s, 2H), 4.46 (s, 2H), 3.80 (s, 3H), 3.52 (s, 3H), 2.91 (s, 3H); ^13^C NMR (75 MHz, CDCl_3_) δ 157.58, 154.44, 151.63, 147.93, 139.74, 136.34, 133.15, 130.31, 128.77, 128.47, 127.84, 127.78, 105.07, 57.44, 43.57, 38.91, 32.90, 29.72 ppm. LCMS R_T_ = 5.59 min; HRMS, calc’d for C_22_H_23_ClN_5_O_2_^+^ [M + H], 424.1535; found 424.1540.

1-Benzyl-3,7-dimethyl-3,7-dihydro-1H-purine-2,6-dione (**44**). Theobromine (**10**) (1.58 g, 8.78 mmol, 1.5 eq) and K_2_CO_3_ (1.62 g, 11.7 mmol, 2.0 eq) were suspended in DMF (8.0 mL), followed by the addition of benzyl bromide (1.00 g, 5.85 mmol, 1.0 eq). The reaction was heated at 90 °C overnight. The reaction was cooled to room temperature, water was added, and the residue was filtered and rinsed with water. The product was dried at 40 °C to provide 1.50 g (95%) of the title compound as a white powder that was used further without purification. LCMS R_T_ = 3.98 min; HRMS, calc’d for C_14_H_15_N_4_O_2_^+^ [M + H], 271.1190; found 271.1192.

1-Benzyl-8-chloro-3,7-dimethyl-3,7-dihydro-1H-purine-2,6-dione (**45**). Intermediate **44** (1.50 g, 5.55, 1.0 eq) and NCS (1.48 g, 11.1 mmol, 2.0 eq) were suspended in THF (15 mL), and the reaction was heated at 60 °C overnight. THF was removed in vacuo, and the residue was filtered and washed with water. The product was dried at 40 °C to provide 1.31 g (78%) of the title compound as a beige solid that was used further without purification. ^1^H NMR (300 MHz, CDCl_3_) δ 7.47 (m, 2H), 7.40–7.18 (m, 3H), 5.18 (s, 2H), 3.95 (s, 3H), 3.53 (s, 3H). LCMS R_T_ = 4.64 min; HRMS, calc’d for C_14_H_14_ClN_4_O_2_^+^ [M + H], 305.0800; found 305.0799.

1-Benzyl-8-(benzyl(methyl)amino)-3,7-dimethyl-3,7-dihydro-1H-purine-2,6-dione [VU0607689] (**8**). Intermediate **45** (50 mg, 0.16 mmol, 1.0 eq), benzyl amine (34 mg, 0.32 mmol, 2.0 eq), Cs_2_CO_3_ (104 mg, 0.32 mmol, 2.0 eq), and DMF (0.5 mL) were added to a microwave vial and heated in a microwave reactor at 130 °C for 30 min. The reaction was cooled to room temperature, water was added, and the mixture was extracted with ethyl acetate (2×). The combined organics were washed with brine, dried over Na_2_SO_4_, filtered, and concentrated in vacuo. Purification of the residue by flash chromatography on silica gel yielded impure product that was dissolved in THF (2.0 mL) and cooled to 0 °C, followed by the addition of sodium hydride (15 mg, 0.64 mmol, 4.0 eq). The solution was stirred for 30 min, followed by the addition of methyl iodide. The reaction temperature was brought to 50 °C and stirred at that temperature for 1 h. The reaction was cooled to room temperature, water was added, and the mixture was extracted with DCM (2×). The combined organics were washed with brine, dried over Na_2_SO_4_, filtered, and concentrated in vacuo. Purification of the residue by flash chromatography on silica gel yielded 20 mg (32%) of the title compound. ^1^H NMR (300 MHz, CDCl_3_) δ 7.49 (m, 2H), 7.41–7.18 (m, 8H), 5.19 (s, 2H), 4.45 (s, 2H), 3.80 (s, 3H), 3.52 (s, 3H), 2.90 (s, 3H); ^13^C NMR (75 MHz, CDCl_3_) δ 157.49, 154.61, 151.70, 147.83, 137.83, 136.79, 128.76, 128.74, 128.35, 127.86, 127.76, 127.32, 105.14, 57.48, 44.22, 38.93, 32.86, 29.70 ppm. LCMS R_T_ = 5.26 min; HRMS, calc’d for C_22_H_24_N_5_O_2_^+^ [M + H], 390.1925; found 390.1932.

1-Benzyl-8-(benzylamino)-3,7-dimethyl-3,7-dihydro-1H-purine-2,6-dione (**46a**). Intermediate **45** (700 mg, 2.30 mmol, 1.0 eq), benzyl amine (493 mg, 4.60 mmol, 2.0 eq), Cs_2_CO_3_ (1.50 g, 4.60 mmol, 2.0 eq), and DMF (5.0 mL) were added to a microwave vial and heated in a microwave reactor at 130 °C for 30 min. The reaction was cooled to room temperature, water was added, and the mixture was extracted with ethyl acetate (2×). The combined organics were washed with brine, dried over Na_2_SO_4_, filtered, and concentrated in vacuo. Purification of the residue by flash chromatography on silica gel yielded 650 mg (38%) of the title compound. ^1^H NMR (300 MHz, CDCl_3_) δ 7.47 (m, 2H), 7.41–7.16 (m, 8H), 5.18 (s, 2H), 4.66 (d, J = Hz, 2H), 4.40 (m, 1H), 3.66 (s, 3H), 3.52 (s, 3H); ^13^C NMR (75 MHz, CDCl_3_) δ 154.12, 153.21, 151.69, 148.73, 138.13, 137.92, 128.87, 128.61, 128.34, 127.98, 127.26, 103.37, 47.43, 44.11, 29.72, 29.63 ppm. LCMS R_T_ = 4.87 min; HRMS, calc’d for C_21_H_22_N_5_O_2_^+^ [M + H], 376.1768; found 376.1774.

1-Benzyl-8-(benzyl(ethyl)amino)-3,7-dimethyl-3,7-dihydro-1H-purine-2,6-dione (**67**). Compound **46a** (50 mg, 0.13 mmol, 1.0 eq) was dissolved in THF (2.0 mL) and cooled to 0 °C, followed by the addition of NaH (12.5 mg, 0.52 mmol, 4.0 eq). The solution was stirred for 30 min, followed by the addition of iodoethane. The reaction temperature was brought to 50 °C and stirred for 1 h. The reaction was cooled to room temperature, water was added, and the mixture was extracted with DCM (2×). The combined organics were washed with brine, dried over Na_2_SO_4_, filtered, and concentrated in vacuo. Purification of the residue by flash chromatography on silica gel yielded 21 mg (40%) of the title compound. ^1^H NMR (300 MHz, CDCl_3_) δ 7.49 (m, 2H), 7.39–7.18 (m, 8H), 5.18 (s, 2H), 4.54 (s, 2H), 3.76 (s, 3H), 3.51 (s, 3H), 3.27 (q, J = 7.11 Hz, 2H), 1.15 (t, J = 7.11 Hz, 3H); ^13^C NMR (75 MHz, CDCl_3_) δ 156.56, 154.62, 151.69, 147.78, 137.83, 137.44, 128.77, 128.62, 128.34, 127.95, 127.59, 127.32, 105.10, 54.71, 46.00, 44.21, 32.58, 29.71, 12.68 ppm. LCMS R_T_ = 5.51 min; HRMS, calc’d for C_23_H_26_N_5_O_2_^+^ [M + H], 404.2081; found 404.2088.

### 3.3. Stable Cell Line Generation

Stably transfected monoclonal HEK293 cell lines expressing Slick, Maxi-K α1/β3, Maxi-K α1/β4, Ca_V_3.2, GIRK 1/2, K_V_2.1, K_V_7.2, Na_V_1.7, and TASK-1 were generated as previously described [9]. Stably transfected monoclonal HEK293 cell lines expressing hERG were generated as previously described [66]. CHO-K1 cells expressing human Slack and disease-associated human Slack mutations, G288S and A934T, were generated by transfecting a PiggyBac TRE mammalian expression vector (VectorBuilder, Chicago, IL, USA) containing KCNT1. FuGENE 6 (Promega, Madison, WI, USA) transfection reagent and protocol, together with Opti-MEM (Thermo Fisher Scientific, Walkham, MA, USA), were utilized.

### 3.4. Whole-Cell Automated Patch-Clamp Electrophysiology

Automated patch-clamp recording was performed with a SyncroPatch 768 PE system (Nanion, Munich, Germany) as previously described [9,67]. Whole-cell recordings were performed at room temperature. Cells were harvested by first washing with PBS without Mg^2+^ or Ca^2+^ (Gibco, Billings, MT, USA) to remove cell culture media, followed by dissociation with TryPLE (Gibco, Billings, MT, USA). Cells were then resuspended and diluted to 100,000 cells/mL in external solution. External solution contained 105 mM NaCl, 4 mM KCl, 1 mM MgCl_2_, 5 mM CaCl_2_, 10 mM HEPES, and 40 mM N-Methyl-D-glucamine chloride, titrated to pH of 7.4 and osmolarity of 300 mOsm/kg; internal solution contained 70 mM NaCl, 70 mM KF, 10 mM KCl, 5 mM EGTA, and 5 mM HEPES, titrated to a pH of 7.2 and osmolarity of 295 mOsm/kg. Compound potencies were generated using concentrations ranging from 0.125 to 30 μM in half-log steps and a minimum of three cells (replicates) per concentration series. 

The APC protocol was set as follows: Cells were held at a pressure of −80 mV for 100 ms, then a ramp protocol was performed from −100 mV to 80 mV at 0.4 mV/ms, followed by holding the cell at −80 mV for another 100 ms, then steps were performed at −20 mV, 0 mV, +20 mV, +40 mV, and +60 mV for 100 ms of each step. Whole-cell currents were measured at 0 mV and normalized to current after external buffer addition. Compound response was compared to “full block” E_max_ of 500 μM quinidine. To obtain the concentration-response relationship graphs and IC_50_ values, we take the median value of the last 7 current amplitude values obtained for each set of data points collected for a given concentration at a particular step.

### 3.5. Thallium (Tl^+^)-Flux Assays

The Tl^+^ flux assays were conducted as previously described [9,60] with modifications optimized for individual cell lines as described below. Following removal of cell culture media, cells were loaded with Tl^+^-sensitive dye loading solution containing Thallos-AM (ION Biosciences, San Marcos, TX, USA), 1.25 mM Probenecid, and 0.3% Pluronic F-127 (MilliporeSigma, Burlington, MA, USA), in assay buffer comprised of Hanks Balanced Salt Solution (HBSS) (Thermo Fisher Scientific, Waltham, MA, USA) and 20 mM HEPES (Corning, Corning, NY, USA) at room temperature for one-hour. Dye loading solution was replaced with 20 μL/well of assay buffer prior to assaying. Cell plates were then loaded onto a Panoptic kinetic imaging plate reader (WaveFront Biosciences, Franklin, TN, USA). Data were acquired at 5 Hz (excitation 482 ± 35 nm, emission 536 ± 40 nm) for 10 s, at which time 20 μL/well of test compounds in assay buffer at 2-fold above the desired final concentration was added and allowed to incubate for 120 s. Next, 10 μL/well of Tl^+^ stimulus buffer (129 mM Na gluconate, 1.2 mM CaSO_4_, 1 MgSO_4_, 1 mM Na_2_HPO_4_, 4.17 mM NaHCO_3_, 5.56 mM glucose, 2.5 mM Tl_2_SO_4_, and 10 mM HEPES pH 7.3) was added. Imaging was concluded after an additional 120 s. Compound potencies were determined using concentrations ranging from 1.5 nm to 30 μM in half-log steps and a minimum of three replicate wells per concentration series.

## Figures and Tables

**Table 1:** Eastern ring analogs.

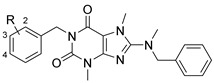			
Analog	R	SLACK IC_50_ (µM) ^1^	SLACK I_max_^1,2^
**8**	H	1.7	100
**16**	2-F	1.8	100
**17**	3-F	1.6	100
**18**	4-F	1.3	100
**19**	2-Cl	2.0	89
**20**	3-Cl	1.9	100
**21**	4-Cl	2.6	100
**22**	2-CH_3_	1.6	100
**23**	3-CH_3_	1.5	100
**24**	4-CH_3_	5.5	102
**25**	2-CF_3_	>10	70
**26**	3-CF_3_	4.2	100
**27**	4-CF_3_	9.7	79
**28**	2-CN	2.5	99
**29**	3-CN	2.4	100
**30**	4-CN	2.4	100
**31**	2-OCH_3_	1.3	100
**32**	3-OCH_3_	0.80	100
**33**	4-OCH_3_	>10	78

^1^ Concentration-response relationships obtained from whole-cell automated patch-clamp (SyncroPatch 384 from Nanion Technologies GmbH) in CHO cells stably expressing WT hSLACK. Values represent averages (*n* ≥ 3). ^2^ Maximum inhibition at 30 µM test compound standardized to VU0606170 (set at 100%).

**Table 2:** *N*^7^-position analogs.

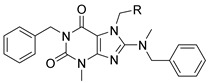			
Analog	R	SLACK IC_50_ (µM) ^1^	SLACK I_max_^1,2^
**41**	CH_3_	>10	87
**42**	CH_2_CH_3_	>10	94
**43**	C_3_H_5_	>10	55

^1^ Concentration-response relationships obtained from whole-cell automated patch-clamp (SyncroPatch 384 from Nanion Technologies GmbH) in CHO cells stably expressing WT hSLACK. Values represent averages (*n* ≥ 3). ^2^ Maximum inhibition at 30 µM test compound standardized to VU0606170 (set at 100%).

**Table 3:** Western ring analogs.

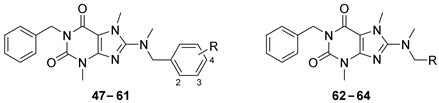			
Analog	R	SLACK IC_50_ (µM) ^1^	SLACK I_max_^1,2^
**47**	2-F	2.7	100
**48**	3-F	0.89	100
**49**	4-F	1.6	100
**50**	2-Cl	3.0	100
**51**	3-Cl	1.7	100
**52**	4-Cl	2.3	100
**53**	2-CH_3_	9.9	100
**54**	3-CH_3_	2.0	100
**55**	4-CH_3_	1.8	100
**56**	2-CF_3_	1.9	100
**57**	3-CF_3_	1.5	101
**58**	4-CF_3_	1.9	100
**59**	2-OCH_3_	4.7	101
**60**	3-OCH_3_	2.7	100
**61**	4-OCH_3_	2.4	100
**62**	pyridin-2-yl	>10	107
**63**	pyridin-3-yl	>10	20
**64**	pyridin-4-yl	>10	70

^1^ Concentration-response relationships obtained from whole-cell automated patch-clamp (SyncroPatch 384 from Nanion Technologies GmbH) in CHO cells stably expressing WT hSLACK. Values represent averages (*n* ≥ 3). ^2^ Maximum inhibition at 30 µM test compound standardized to VU0606170 (set at 100%).

**Table 4:** Western linker analogs.

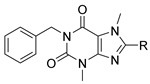			
Analog	R	SLACK IC_50_ (µM) ^1^	SLACK I_max_^1,2^
**46a**	N(H)CH_2_Ph	6.0	88
**65**	(*R*)-N(Me)CH(Me)Ph	1.6	100
**66**	(*S*)-N(Me)CH(Me)Ph	>10	50
**67**	N(Et)CH_2_Ph	1.4	100
**68**	N(*n*-Pr)CH_2_Ph	1.3	100
**69**	N(*i*-Pr)CH_2_Ph	3.4	100
**70**	N(*n*-Bu)CH_2_Ph	0.94	100
**71**	N(CH_2_C_3_H_5_)CH_2_Ph	0.98	100
**72**	N(CH_2_Ph)_2_	1.4	100
**73**	OCH_2_Ph	Not Determined	
**74**	isoindolin-2-yl	Not Determined	
**75**	3,4-dihydroisoquinolin-2(1*H*)-yl	2.8	100
**76**	C≡CPh	Not Determined	
**77**	CH_2_CH_2_Ph	7.0	103

^1^ Concentration-response relationships obtained from whole-cell automated patch-clamp (SyncroPatch 384 from Nanion Technologies GmbH) in CHO cells stably expressing WT hSLACK. Values represent averages (*n* ≥ 3). ^2^ Maximum inhibition at 30 µM test compound standardized to VU0606170 (set at 100%).

**Table 5:** Ion-channel panel screening with selected analogs ^1^.

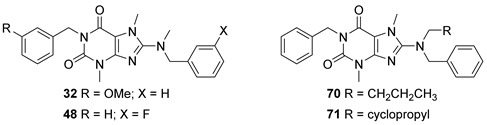				
	Percent Inhibition ± SEM ^2^			
Target	32	48	70	71
SLICK	28 ± 5.5	21 ± 3.1	18 ± 4.2	17 ± 2.5
Maxi-K α1/β3	79 ± 1.4	63 ± 1.2	34 ± 2.9	17 ± 5.2
Maxi-K α1/β4	48 ± 2.0	42 ± 4.6	8.5 ± 2.1	12 ± 3.9
Ca_V_3.2	18 ± 1.6	17 ± 4.2	16 ± 2.2	24 ± 3.6
GIRK 1/2	19 ± 1.7	32 ± 3.0	9.2 ± 1.4	12 ± 0.5
hERG	4.6 ± 4.5	−2.8 ± 2.4	22 ± 2.0	23 ± 4.0
K_V_2.1	51 ± 1.5	51 ± 1.2	35 ± 1.3	33 ± 1.2
K_V_7.2	16 ± 2.2	15 ± 2.3	19 ± 0.2	14 ± 1.6
Na_V_1.7	41 ± 3.3	39 ± 3.1	33 ± 1.4	29 ± 3.0
TASK-1	17 ± 2.3	24 ± 2.3	23 ± 2.9	4.9 ± 1.5

^1^ Obtained with 10 µM test compound via Tl^+^-flux assay in HEK-293 cells stably expressing target. ^2^ Percent inhibition relative to the following control compounds: SKF96365 (SLICK), paxilline (Maxi-K), bepridil (Ca_V_3.2, hERG, TASK1), SCH23390 (GIRK 1/2), guangxitoxin 1E (K_V_2.1), XE991 (K_V_7.2), and TC-N 1752 (Na_V_1.7).

**Table 6:** Electrophysiology results versus mouse and GOF mutant SLACK.

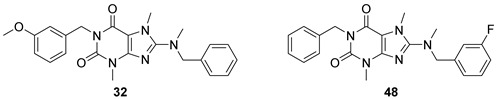			
Analog	Target	IC_50_ (µM) ^1^	I_max_^1,2^
	mouse WT	1.4	100
**32**	human A934T	0.78	99
	human G288S	0.65	100
	mouse WT	1.3	100
**48**	human A934T	0.82	100
	human G288S	0.75	100

^1^ Concentration-response relationships obtained from whole-cell automated patch-clamp (SyncroPatch 384 from Nanion Technologies GmbH) in CHO cells stably expressing target. ^2^ Maximum inhibition at 30 µM test compound standardized to VU0606170 (set at 100%).

**Table 7:** Combination analogs: electrophysiology results versus WT and GOF mutant SLACK.

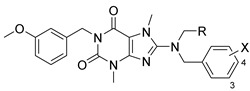					
			IC_50_ (µM) ^1^		
Analog	R	X	WT SLACK	A934T	G288S
**78**	CH_2_CH_2_CH_3_	3-F	1.2	0.35	0.88
**79**	C_3_H_5_	3-F	1.0	1.0	1.0
**80**	CH_2_CH_2_CH_3_	4-F	0.70	0.59	0.71
**81**	C_3_H_5_	4-F	1.5	1.2	0.79

^1^ Concentration-response relationships obtained from whole-cell automated patch-clamp (SyncroPatch 384 from Nanion Technologies GmbH) in CHO cells stably expressing WT hSLACK.

## Data Availability

Data supporting reported results is available upon request.

## Supplementary Materials

The following supporting information can be downloaded at: https://www.mdpi.com/article/10.3390/molecules29112437/s1, Synthetic methods and characterization of all samples submitted for biological testing.
