# Supplementary material for: Structure–Activity Relationship Studies in a Series of Xanthine Inhibitors of SLACK Potassium Channels
Source: Molecules. 2024 May 22;29(11):2437. doi: 10.3390/molecules29112437 (PMC11173529; doi:10.3390/molecules29112437)
Supplement: Supplementary file 1 [file molecules-29-02437-s001.zip › molecules-3013059-supplementary.pdf]

Structure-Activity Relationship Studies in a Series of Xanthine Inhibitors of SLACK Potassium Channels

Alshaima'a M. Qunies [1,2], Brittany D. Spitznagel [3], Yu Du [3,4], Paul K. Peprah [1,2], Yasmeen K. Mohamed [1], C. David Weaver [3,4], and Kyle A. Emmitte [1,*]

[1] Department of Pharmaceutical Sciences, UNT System College of Pharmacy, University of North Texas Health Science Center, Fort Worth, TX 76107 USA

[2] School of Biomedical Sciences, University of North Texas Health Science Center, Fort Worth, TX 76107 USA

[3] Department of Pharmacology, Vanderbilt University, Nashville, TN 37232 USA

[4] Vanderbilt Institute for Chemical Biology, Vanderbilt University, Nashville, TN 37232 USA

* Correspondence: kyle.emmitte@unthsc.edu; Tel.: +1 817 735 0241

Synthesis and Purification

Air-sensitive reactions were carried out under a nitrogen atmosphere. Starting materials, reagents, intermediates, and final compounds were weighed on a Mettler Toledo™ New Classic ME analytical balance or a Mettler Toledo™ New Classic ME toploader balance. Thin-layer chromatography (TLC) was conducted on glass plates coated with Silica Gel 60 F_{254} from Millipore Sigma. Normal-phase flash chromatography was carried out on either a CombiFlash® EZ Prep or CombiFlash® Rf+ automated flash chromatography system, both from Teledyne ISCO. Normal-phase flash chromatography was carried out using Redi*Sep*® Rf normal-phase, disposable flash columns from Teledyne ISCO or SiliaSep normal-phase, disposable flash columns (40-63 micron) from SiliCycle, Inc. Reverse-phase preparative chromatography was carried out on the CombiFlash® EZ Prep using a reusable Redi*Sep*® Rf C18 reverse-phase column. Microwave reactions were carried out an Anton Paar Monowave 200 automated microwave synthesizer. The Monowave 200 has an output power of 850W with a maximum temperature of 260 °C and a maximum pressure of 290 psi and is suitable for use with reaction volumes ranging from 0.5 to 20 mL.

All NMR spectra were recorded on a 300 MHz Bruker Fourier 300HD NMR spectrometer equipped with a dual ^{1}H and ^{13}C probe with Z-Gradient and automatic tuning and matching, full computer control of all shims with TopShim™, 24-sample SampleCase™

automation system, and TopSpin™ software. All NMR samples were prepared with chloroform-d with 0.03% TMS (99.8+ atom % D, Acros Organics Catalog No. 209561000) or d_6-dimethyl sulfoxide with 0.03% TMS (ACROS Organics Catalog No. 360000100). ^{1}H and ^{13}C chemical shifts are reported in δ values in ppm downfield. Data are reported as follows: chemical shift, multiplicity (s = singlet, d = doublet, t = triplet, q = quartet, br = broad, m = multiplet), integration, coupling constant (Hz). High resolution mass spectrometry was conducted on an Agilent 6230 Accurate-Mass Time-of-Flight (TOF) LC/MS with ESI source equipped with MassHunter Walkup software. MS parameters were as follows: fragmentor: 175 V, capillary voltage: 3500 V, nebulizer pressure: 35 psig, drying gas flow: 11 L/min, drying gas temperature: 325 °C. Samples were introduced via an Agilent 1260 Infinity UHPLC comprised of a G4225A HiP Degasser, G1312B binary pump, G1367E ALS, G1316A TCC, and G1315C DAD VL+ with a 5 μL semi-micro flow cell with a 6 mm path length. UV absorption was observed at 220 nm and 254 nm with a 4 nm bandwidth. Column: Agilent Zorbax SB-C18, Rapid Resolution HT, 1.8 μm, 2.1 x 50 mm. Gradient conditions: Hold at 5% CH_3CN in H_2O (0.1% formic acid) for 1.0 min, 5% to 95% CH_3CN in H_2O (0.1% formic acid) over 5 min, hold at 95% CH_3CN in H_2O (0.1% formic acid) for 1.0 min, 0.5 mL/min. All samples submitted for biological testing were confirmed ≥ 95% pure by ^{1}H NMR.

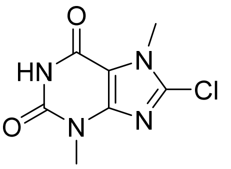

8-Chloro-3,7-dimethyl-3,7-dihydro-1*H*-purine-2,6-dione (14). Theobromine (**10**) (5.00 g, 27.8 mmol, 1.0 eq), and NCS (7.41 g, 55.5 mmol, 2.0 eq) were suspended in THF (25 mL), and the reaction was heated at 60 °C overnight. THF was removed *in vacuo*, and the residue was filtered and washed with water. The product was dried at 40 °C to provide 4.4 g (74%) of the title compound as a beige solid that was used further without purification. LCMS R_T = 2.62 min; HRMS, calc'd for $C_7H_8ClN_4O_2^+$ [M+H], 215.0330; found 215.0330.

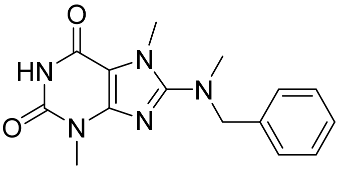

8-(Benzyl(methyl)amino)-3,7-dimethyl-3,7-dihydro-1*H*-purine-2,6-dione (15). Intermediate **14** (1.00 g, 4.66 mmol, 1.0 eq), N-methylbenzyl amine (1.13 g, 9.32 mmol, 2.0 eq), Cs_2CO_3 (3.03 g, 9.32 mmol, 2.0 eq), and DMF (5.0 mL) were added to a microwave vial and heated in a microwave reactor at 130 °C for 30 minutes. The reaction was cooled to room temperature, water was added, and the mixture was extracted with ethyl acetate (2x). The combined organics were washed with brine, dried over Na_2SO_4, filtered, and concentrated *in vacuo*. Purification of the residue by flash chromatography on silica gel

yielded 600 mg (43%) of the title compound. LCMS R_T = 4.0 min; HRMS, calc'd for $C_{15}H_{18}N_5O_2^+$ [M+H], 300.1455; found 300.1460.

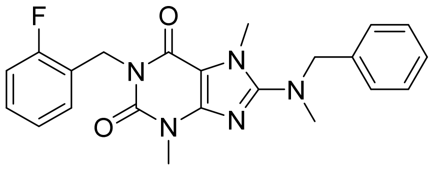

8-(Benzyl(methyl)amino)-1-(2-fluorobenzyl)-3,7-dimethyl-3,7-dihydro-1*H*-purine-2,6-dione (16). Sodium hydride (8.0 mg, 0.34 mmol, 2.0 eq) was added to an ice-cold solution of **15** (50 mg, 0.17 mmol, 1.0 eq), and was stirred for 15 minutes. Afterwards, 2-fluorobenzyl methanesulfonate (69 mg, 0.34 mmol, 2.0 eq) was added, and the reaction was heated at 130 °C for one hour. The reaction was cooled to room temperature, water was added, and the mixture was extracted with DCM (2x). The combined organics were washed with brine, dried over Na_2SO_4, filtered, and concentrated *in vacuo*. Purification of the residue by flash chromatography on silica gel yielded 25 mg (36%) of the title compound. ^{1}H NMR (300 MHz, $CDCl_3$) δ 7.43 – 7.14 (m, 7H), 7.09 – 6.97 (m, 2H), 5.28 (s, 2H), 4.47 (s, 2H), 3.80 (s, 3H), 3.54 (s, 3H), 2.91 (s, 3H); ^{13}C NMR (75 MHz, $CDCl_3$) δ 162.33, 159.05, 157.58, 154.43, 151.61, 148.00, 136.77, 128.86 (d, *J*(C,F) = 4.07 Hz), 128.64 (d, *J*(C,F) = 8.29 Hz), 128.31 (d, *J*(C,F) = 69.62 Hz), 127.78, 124.68 (d, *J*(C,F) = 14.40 Hz), 124.00 (d, *J*(C,F) = 3.65 Hz), 115.35 (d, *J*(C,F) = 21.73 Hz), 105.03, 57.45, 38.91, 38.00 (d, *J*(C,F) = 4.97

Hz), 32.92, 29.76 ppm. LCMS R_T = 5.25 min; HRMS, calc'd for $C_{22}H_{23}FN_5O_2^+$ [M+H], 408.1830; found 408.1836.

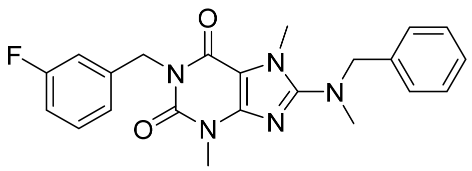

8-(Benzyl(methyl)amino)-1-(3-fluorobenzyl)-3,7-dimethyl-3,7-dihydro-1*H*-purine-2,6-dione (17). 3-Fluorobenzyl 4-methylbenzenesulfonate (95.3 mg, 0.34 mmol, 2.0 eq) was added to a solution of **15** (50 mg, 0.17 mmol, 1.0 eq) and Cs_2CO_3 (110 mg, 0.34 mmol, 2.0 eq) in THF (3 mL), and stirred at 60 °C overnight. The reaction was cooled to room temperature, water was added, and the mixture was extracted with DCM (2x). The combined organics were washed with brine, dried over Na_2SO_4, filtered, and concentrated *in vacuo*. Purification of the residue by flash chromatography on silica gel yielded 15 mg (22%) of the title compound. ^{1}H NMR (300 MHz, $CDCl_3$) δ 7.41 – 7.21 (m, 7H), 7.17 (m, 1H), 6.93 (m, 1H), 5.17 (s, 2H), 4.47 (s, 2H), 3.81 (s, 3H), 3.53 (s, 3H), 2.91 (s, 3H); ^{13}C NMR (75 MHz, $CDCl_3$) δ 162.77 (d, *J*(C,F) = 245.42 Hz), 157.60, 154.42, 151.63, 147.97, 140.25 (d, *J*(C,F) = 7.40 Hz), 136.75, 129.79 (d, *J*(C,F) = 8.25 Hz), 128.77, 127.84, 127.78, 124.21 (d, *J*(C,F) = 2.80 Hz), 115.41 (d, *J*(C,F) = 21.82 Hz), 114.23 (d, *J*(C,F) = 21.04 Hz), 105.05, 57.44, 42.74 (d, *J*(C,F) = 1.67 Hz), 38.91, 32.91, 29.74 ppm. LCMS R_T = 5.33 min; HRMS, calc'd for $C_{22}H_{23}FN_5O_2^+$ [M+H], 408.1830; found 408.1835.

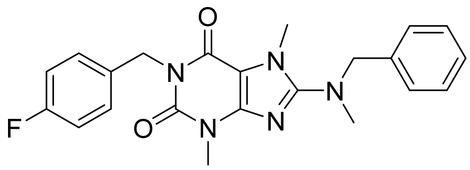

8-(Benzyl(methyl)amino)-1-(4-fluorobenzyl)-3,7-dimethyl-3,7-dihydro-1*H*-purine-2,6-dione (18). 4-Fluorobenzylchloride (49 mg, 0.34 mmol, 2.0 eq) was added to a solution of **15** (50 mg, 0.17 mmol, 1.0 eq) and Cs_2CO_3 (110 mg, 0.34 mmol, 2.0 eq) in DMF (0.5 mL), and stirred at 100 °C overnight. The reaction was cooled to room temperature, water was added, and the mixture was extracted with DCM (2x). The combined organics were washed with brine, dried over Na_2SO_4, filtered, and concentrated *in vacuo*. Purification of the residue by flash chromatography on silica gel yielded 28 mg (40%) of the title compound. ^{1}H NMR (300 MHz, $CDCl_3$) δ 7.49 (m, 2H), 7.41 – 7.27 (m, 5H), 6.97 (m, 2H), 5.14 (s, 2H), 4.46 (s, 2H), 3.80 (s, 3H), 3.52 (s, 3H), 2.90 (s, 3H); ^{13}C NMR (75 MHz, $CDCl_3$) δ 162.18 (d, *J*(C,F) = 245.07 Hz), 157.55, 154.50, 151.65, 147.88, 136.75, 133.65 (d, *J*(C,F) = 3.20 Hz), 130.74 (d, *J*(C,F) = 8.16 Hz), 128.76, 127.84, 127.77, 115.11 (d, *J*(C,F) = 21.19 Hz), 105.10, 57.45, 43.49, 38.92, 32.88, 29.71 ppm. LCMS R_T = 5.38 min; HRMS, calc'd for $C_{22}H_{23}FN_5O_2^+$ [M+H], 408.1830; found 408.1830.

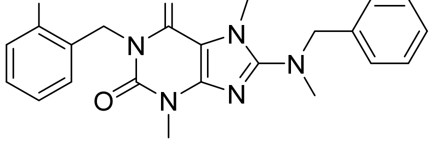

8-(Benzyl(methyl)amino)-1-(2-chlorobenzyl)-3,7-dimethyl-3,7-dihydro-1*H*-purine-2,6-dione (19). The title compound was prepared in 22% yield from intermediate **15** and 2-chlorobenzyl chloride using a method analogous to that described for the conversion of intermediate **15** into compound **18**. ^{1}H NMR (300 MHz, $CDCl_3$) *δ* 7.44 – 7.28 (m, 6H), 7.15 (m, 2H), 7.00 (m, 1H), 5.32 (s, 2H), 4.49 (s, 2H), 3.81 (s, 3H), 3.56 (s, 3H), 2.94 (s, 3H); ^{13}C NMR (75 MHz, $CDCl_3$) δ 157.65, 154.39, 151.60, 148.12, 136.76, 134.91, 132.92, 129.51, 128.77, 128.01, 127.85, 127.80, 126.82, 126.68, 104.99, 57.44, 41.99, 38.91, 32.95, 29.81 ppm. LCMS R_T = 5.52 min; HRMS, calc'd for $C_{22}H_{23}ClN_5O_2^+$ [M+H], 424.1535; found 424.1549.

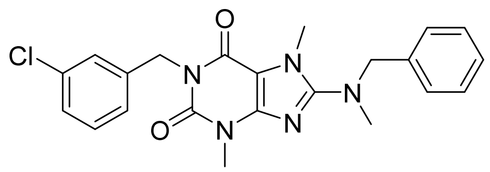

8-(Benzyl(methyl)amino)-1-(3-chlorobenzyl)-3,7-dimethyl-3,7-dihydro-1*H*-purine-2,6-dione (20). The title compound was prepared in 20% yield from intermediate **15** and 3-chlorobenzyl chloride using a method analogous to that described for the conversion of intermediate **15** into compound **18**. ^{1}H NMR (300 MHz, $CDCl_3$) *δ* 7.44 (m, 1H), 7.41 – 7.18 (m, 8H), 5.15 (s, 2H), 4.47 (s, 2H), 3.81 (s, 3H), 3.53 (s, 3H), 2.91 (s, 3H); ^{13}C NMR (75 MHz, $CDCl_3$) δ 157.61, 154.39, 151.61, 147.98, 139.78, 136.75, 134.17, 129.59, 128.77, 128.55, 127.84, 127.78, 127.54, 126.90, 105.05, 57.44, 43.71, 38.92, 32.92, 29.75 ppm. LCMS R_T = 5.62 min; HRMS, calc'd for $C_{22}H_{23}ClN_5O_2^+$ [M+H], 424.1535; found 424.1545.

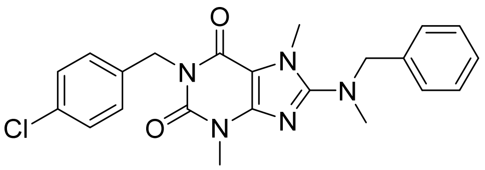

8-(Benzyl(methyl)amino)-1-(4-chlorobenzyl)-3,7-dimethyl-3,7-dihydro-1*H*-purine-2,6-dione (21). 4-Chlorobenzyl bromide (33 mg, 0.16 mmol, 2.0 eq) was added to a solution of **15** (25 mg, 0.08 mmol, 1.0 eq) and K_2CO_3 (22 mg, 0.16 mmol, 2.0 eq) in DMF (0.5 mL), and stirred at 100 °C overnight. The reaction was cooled to room temperature, water was added, and the mixture was extracted with DCM (2x). The combined organics were washed with brine, dried over sodium sulfate (Na_2SO_4), filtered, and concentrated *in vacuo*. Purification of the residue by flash chromatography on silica gel yielded 24 mg (71%) of the title compound. ^{1}H NMR (300 MHz, $CDCl_3$) δ 7.43 (m, 2H), 7.40 – 7.21 (m, 7H), 5.13 (s, 2H), 4.46 (s, 2H), 3.80 (s, 3H), 3.52 (s, 3H), 2.91 (s, 3H); ^{13}C NMR (75 MHz, $CDCl_3$) δ 157.58, 154.44, 151.63, 147.93, 139.74, 136.34, 133.15, 130.31, 128.77, 128.47, 127.84, 127.78, 105.07, 57.44, 43.57, 38.91, 32.90, 29.72 ppm. LCMS R_T = 5.59 min; HRMS, calc'd for $C_{22}H_{23}ClN_5O_2^+$ [M+H], 424.1535; found 424.1540.

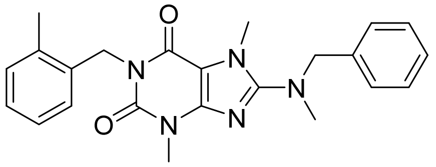

8-(Benzyl(methyl)amino)-3,7-dimethyl-1-(2-methylbenzyl)-3,7-dihydro-1*H*-purine-2,6-dione (22). The title compound was prepared in 28% yield from intermediate **15** and 2-methylbenzyl chloride using a method analogous to that described for the conversion of intermediate **15** into compound **18**. ^{1}H NMR (300 MHz, $CDCl_3$) *δ* 7.44 – 7.30 (m, 5H), 7.19 – 6.96 (m, 4H), 5.19 (s, 2H), 4.48 (s, 2H), 3.81 (s, 3H), 3.55 (s, 3H), 2.92 (s, 3H), 2.47 (s, 3H); ^{13}C NMR (75 MHz, $CDCl_3$) δ 157.56, 154.65, 151.72, 147.96, 136.81, 135.58, 135.50, 130.21, 128.78, 127.87, 127.78, 126.78, 126.01, 125.47, 105.10, 57.48, 41.64, 38.93, 32.91, 29.79, 19.34 ppm. LCMS R_T = 5.43 min; HRMS, calc'd for $C_{23}H_{26}N_5O_2^+$ [M+H], 404.2081; found 404.2091.

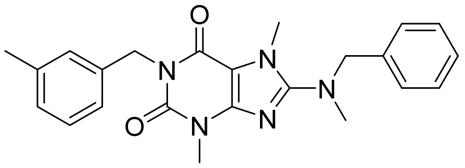

8-(Benzyl(methyl)amino)-3,7-dimethyl-1-(3-methylbenzyl)-3,7-dihydro-1*H*-purine-2,6-dione (23). The title compound was prepared in 39% yield from intermediate **15** and 3-methylbenzyl chloride using a method analogous to that described for the conversion of intermediate **15** into compound **18**. ^{1}H NMR (300 MHz, $CDCl_3$) *δ* 7.43 – 7.23 (m, 7H), 7.18 (m, 1H), 7.04 (m, 1H), 5.15 (s, 2H), 4.45 (s, 2H), 3.81 (s, 3H), 3.53 (s, 3H), 2.90 (s, 3H), 2.32 (s, 3H); ^{13}C NMR (75 MHz, $CDCl_3$) δ 157.47, 154.62, 151.71, 147.82, 137.93, 137.74, 136.80, 129.23, 128.76, 128.24, 128.10, 127.86, 127.76, 125.71, 105.15, 57.49, 44.20, 38.94,

32.87, 29.72, 21.46 ppm. LCMS R_T = 5.48 min; HRMS, calc'd for $C_{23}H_{26}N_5O_2^+$ [M+H], 404.2081; found 404.2090.

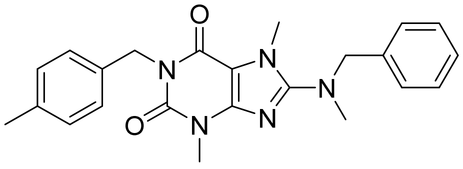

8-(Benzyl(methyl)amino)-3,7-dimethyl-1-(4-methylbenzyl)-3,7-dihydro-1*H*-purine-2,6-dione (24). The title compound was prepared in 44% yield from intermediate **15** and 4-methylbenzyl chloride using a method analogous to that described for the conversion of intermediate **15** into compound **18**. ^1H NMR (300 MHz, $CDCl_3$) δ 7.46 – 7.27 (m, 7H), 7.10 (m, 2H), 5.15 (s, 2H), 4.44 (s, 2H), 3.80 (s, 3H), 3.51 (s, 3H), 2.89 (s, 3H), 2.30 (s, 3H); ^{13}C NMR (75 MHz, $CDCl_3$) δ 157.44, 154.63, 151.69, 147.78, 136.95, 136.79, 134.87, 129.02, 128.81, 128.75, 127.86, 127.76, 105.16, 57.50, 43.96, 38.93, 32.85, 29.69, 21.17 ppm. LCMS R_T = 5.50 min; HRMS, calc'd for $C_{23}H_{26}N_5O_2^+$ [M+H], 404.2081; found 404.2091.

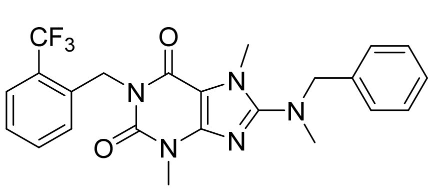

8-(Benzyl(methyl)amino)-3,7-dimethyl-1-(2-(trifluoromethyl)benzyl)-3,7-dihydro-1*H*-purine-2,6-dione (25). The title compound was prepared in 15% yield from intermediate **15** and 2-trifluoromethylbenzyl chloride using a method analogous to that described for

the conversion of intermediate **15** into compound **18**. ^{1}H NMR (300 MHz, $CDCl_3$) *δ* 7.66 (m, 1H), 7.47 – 7.27 (m, 7H), 7.03 (m, 1H), 5.43 (s, 2H), 4.51 (s, 2H), 3.81 (s, 3H), 3.58 (s, 3H), 2.95 (s, 3H); ^{13}C NMR (75 MHz, $CDCl_3$) δ 157.73, 154.36, 151.61, 148.24, 136.74, 136.17, 132.01, 128.80, 127.84, 127.82, 127.68, 127.27, 126.69, 126.10 (q, *J*(C, CF_3) = 5.86 Hz), 125.55, 104.93, 57.42, 41.19, 41.14, 38.90, 32.98, 29.82 ppm. LCMS R_T = 5.61 min; HRMS, calc'd for $C_{23}H_{23}F_3N_5O_2^+$ [M+H], 458.1798; found 458.1833.

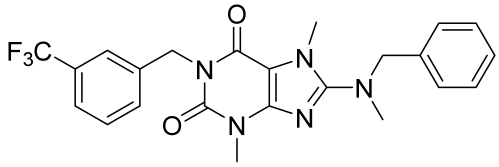

8-(Benzyl(methyl)amino)-3,7-dimethyl-1-(3-(trifluoromethyl)benzyl)-3,7-dihydro-1*H*-purine-2,6-dione (26). The title compound was prepared in 14% yield from intermediate **15** and 3-trifluoromethylbenzyl chloride using a method analogous to that described for the conversion of intermediate **15** into compound **18**. ^{1}H NMR (300 MHz, $CDCl_3$) *δ* 7.77 – 7.62 (m, 2H), 7.55 – 7.27 (m, 7H), 5.23 (s, 2H), 4.48 (s, 2H), 3.81 (s, 3H), 3.54 (s, 3H), 2.92 (s, 3H); ^{13}C NMR (75 MHz, $CDCl_3$) δ 157.65, 154.38, 151.63, 148.02, 138.74, 136.74, 132.19, 130.87, 130.44, 128.77, 127.84, 127.78, 125.42 (q, *J*(C, CF_3) = 3.78 Hz), 124.23 (q, *J*(C, CF_3) = 3.81 Hz), 122.34, 105.03, 57.43, 43.82, 38.90, 32.94, 29.76 ppm. LCMS R_T = 5.70 min; HRMS, calc'd for $C_{23}H_{23}F_3N_5O_2^+$ [M+H], 458.1798; found 458.1797.

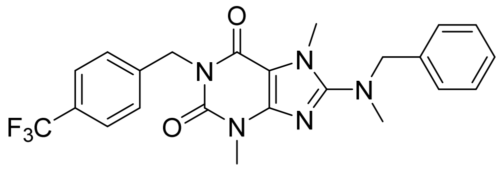

8-(Benzyl(methyl)amino)-3,7-dimethyl-1-(4-(trifluoromethyl)benzyl)-3,7-dihydro-1*H*-purine-2,6-dione (27). The title compound was prepared in 4% yield from intermediate **15** and 4-trifluoromethylbenzyl chloride using a method analogous to that described for the conversion of intermediate **15** into compound **18**. ^{1}H NMR (300 MHz, $CDCl_3$) *δ* 7.57 (m, 4H), 7.34 (m, 5H), 5.22 (s, 2H), 4.47 (s, 2H), 3.80 (s, 3H), 3.53 (s, 3H), 2.91 (s, 3H); ^{13}C NMR (75 MHz, $CDCl_3$) δ 157.67, 154.39, 151.64, 148.04, 141.77, 136.71, 128.88, 127.80, 128.78, 127.83, 125.33 (q, *J*(C, CF_3) = 3.74 Hz), 104.99, 57.42, 43.82, 38.91, 32.93, 29.75 ppm. LCMS R_T = 5.70 min; HRMS, calc'd for $C_{23}H_{23}F_3N_5O_2^+$ [M+H], 458.1798; found 458.1804.

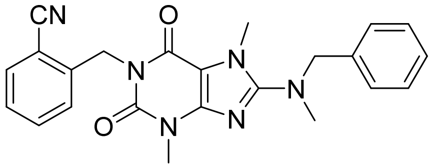

2-((8-(Benzyl(methyl)amino)-3,7-dimethyl-2,6-dioxo-2,3,6,7-tetrahydro-1*H*-purin-1-yl)methyl)benzonitrile (28). The title compound was prepared in 9% yield from intermediate **15** and 2-cyanobenzyl bromide using a method analogous to that described for the conversion of intermediate **15** into compound **21**. ^{1}H NMR (300 MHz, $CDCl_3$) *δ* 7.65 (dd, *J* = 7.71, 0.97 Hz, 1H), 7.48 (td, *J* = 7.74, 0.91 Hz, 1H), 7.42 – 7.27 (m, 7H), 5.42 (s, 2H), 4.49 (s, 2H), 3.81 (s, 3H), 3.54 (s, 3H), 2.93 (s, 3H); ^{13}C NMR (75 MHz, $CDCl_3$) δ 157.75,

154.24, 151.57, 148.26, 141.48, 136.71, 132.93, 132.87, 128.79, 127.83, 127.82, 127.42, 127.16, 117.48, 111.84, 104.90, 57.39, 42.56, 38.88, 33.01, 29.82 ppm. LCMS R_T = 5.09 min; HRMS, calc'd for $C_{23}H_{23}N_6O_2^+$ [M+H], 415.1877; found 415.1891.

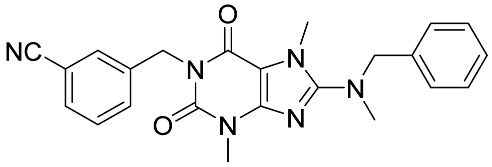

3-((8-(benzyl(methyl)amino)-3,7-dimethyl-2,6-dioxo-2,3,6,7-tetrahydro-1*H*-purin-1-yl)methyl)benzonitrile (29). The title compound was prepared in 39% yield from intermediate **15** and 3-cyanobenzyl bromide using a method analogous to that described for the conversion of intermediate **15** into compound **21**. ^{1}H NMR (300 MHz, $CDCl_3$) δ 7.77 – 7.69 (m, 2H), 7.53 (dt, J = 8.01, 1.40 Hz, 1H), 7.44 – 7.28 (m, 6H), 5.19 (s, 2H), 4.49 (s, 2H), 3.82 (s, 3H), 3.53 (s, 3H), 2.92 (s, 3H); ^{13}C NMR (75 MHz, $CDCl_3$) δ 157.74, 154.23, 151.55, 148.12, 139.30, 136.70, 133.32, 132.09, 131.04, 129.14, 128.78, 127.83, 127.80, 118.90, 112.44, 105.00, 57.38, 43.47, 38.89, 32.97, 29.78 ppm. LCMS R_T = 5.10 min; HRMS, calc'd for $C_{23}H_{23}N_6O_2^+$ [M+H], 415.1877; found 415.1828.

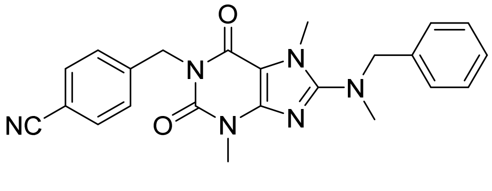

4-((8-(benzyl(methyl)amino)-3,7-dimethyl-2,6-dioxo-2,3,6,7-tetrahydro-1*H*-purin-1-yl)methyl)benzonitrile (30). The title compound was prepared in 18% yield from intermediate **15** and 4-cyanobenzyl bromide using a method analogous to that described for the conversion of intermediate **15** into compound **21**. ^{1}H NMR (300 MHz, $CDCl_3$) δ 7.57 (m, 4H), 7.24 – 7.27 (m, 5H), 5.21 (s, 2H), 4.48 (s, 2H), 3.80 (s, 3H), 3.53 (s, 3H), 2.93 (s, 3H); ^{13}C NMR (75 MHz, $CDCl_3$) δ 157.75, 154.26, 151.59, 148.13, 143.18, 136.67, 132.24, 129.26, 128.79, 127.81, 118.94, 111.14, 104.96, 57.38, 43.91, 38.90, 32.96, 29.77 ppm. LCMS R_T = 5.12 min; HRMS, calc'd for $C_{23}H_{23}N_6O_2^+$ [M+H], 415.1877; found 415.1886.

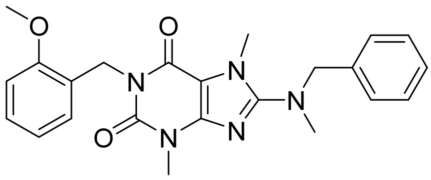

8-(Benzyl(methyl)amino)-1-(2-methoxybenzyl)-3,7-dimethyl-3,7-dihydro-1H-purine-2,6-dione (31). The title compound was prepared in 48% yield from intermediate **15** and 2-methoxylbenzyl chloride using a method analogous to that described for the conversion of intermediate **15** into compound **18**. ^{1}H NMR (300 MHz, $CDCl_3$) δ 7.44 – 7.27 (m, 5H), 7.18 (m, 1H), 6.98 – 6.78 (m, 3H), 5.25 (s, 2H), 4.47 (s, 2H), 3.88 (s, 3H), 3.80 (s, 3H), 3.55 (s, 3H), 2.91 (s, 3H); ^{13}C NMR (75 MHz, $CDCl_3$) δ 157.46, 156.97, 154.66, 151.68, 147.89, 136.85, 128.76, 127.87, 127.75, 126.05, 125.65, 120.43, 110.35, 105.11, 57.50, 55.51,

39.36, 38.93, 32.87, 29.76 ppm. LCMS R_T = 5.27 min; HRMS, calc'd for $C_{23}H_{26}N_5O_3^+$ [M+H], 420.2030; found 420.1955.

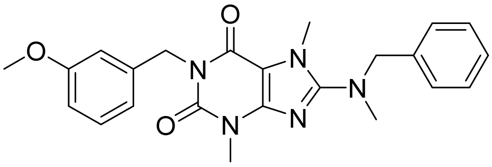

8-(Benzyl(methyl)amino)-1-(3-methoxybenzyl)-3,7-dimethyl-3,7-dihydro-1*H*-purine-2,6-dione (32). The title compound was prepared in 22% yield from intermediate **15** and 3-methoxylbenzyl chloride using a method analogous to that described for the conversion of intermediate **15** into compound **18**. ^{1}H NMR (300 MHz, $CDCl_3$) δ 7.43 – 7.27 (m, 5H), 7.21 (t, J = 7.91 Hz, 1H), 7.10 – 6.97 (m, 2H), 6.78 (m, 1H), 5.17 (s, 2H), 4.46 (s, 2H), 3.79 (d, J = 4.38 Hz, 6H), 3.52 (s, 3H), 2.90 (s, 3H); ^{13}C NMR (75 MHz, $CDCl_3$) δ 159.57, 157.48, 154.55, 151.68, 147.85, 139.37, 136.79, 129.34, 128.76, 127.86, 127.76, 120.84, 114.07, 112.74, 105.11, 57.48, 55.22, 44.14, 38.93, 32.86, 29.72 ppm. LCMS R_T = 5.25 min; HRMS, calc'd for $C_{23}H_{26}N_5O_3^+$ [M+H], 420.2030; found 420.2034.

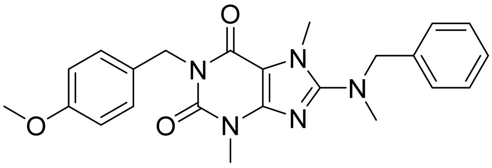

8-(Benzyl(methyl)amino)-1-(4-methoxybenzyl)-3,7-dimethyl-3,7-dihydro-1*H*-purine-2,6-dione (33). The title compound was prepared in 25% yield from intermediate **15** and

4-methoxylbenzyl chloride using a method analogous to that described for the conversion of intermediate **15** into compound **18**. ^{1}H NMR (300 MHz, $CDCl_3$) *δ* 7.47 (m, 2H), 7.42 – 7.27 (m, 5H), 6.82 (m, 2H), 5.12 (s, 2H), 4.44 (s, 2H), 3.78 (d, *J* = 9.77 Hz, 6H), 3.52 (s, 3H), 2.89 (s, 3H); ^{13}C NMR (75 MHz, $CDCl_3$) δ 158.86, 157.44, 154.63, 151.69, 147.76, 136.79, 130.30, 130.13, 128.75, 127.86, 127.75, 113.67, 105.18, 57.49, 55.24, 43.63, 38.93, 32.84, 29.68 ppm. LCMS R_T = 5.24 min; HRMS, calc'd for $C_{23}H_{26}N_5O_3^+$ [M+H], 420.2030; found 420.2060.

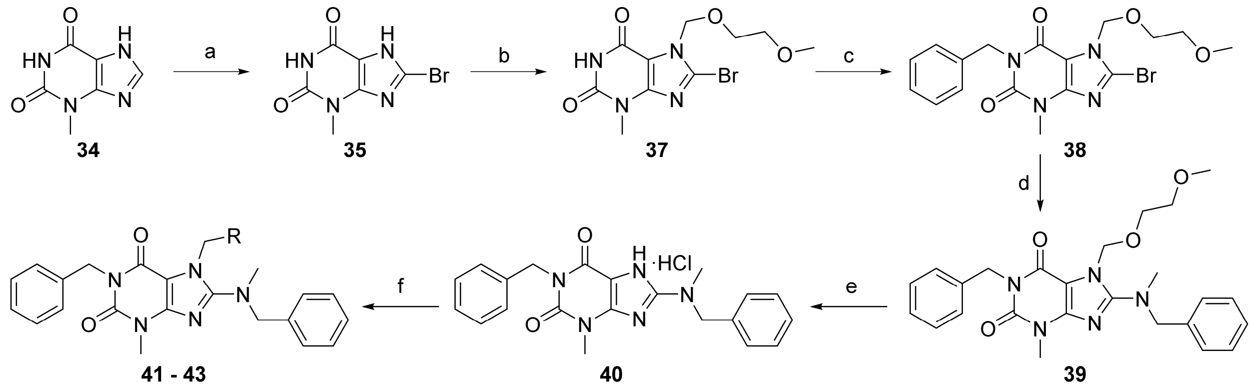

Scheme 2. Synthesis of N^7-position analogs. *Reagents and Conditions:* (a) Br_2, AcOH, 100 °C, *95%*; (b) $ClCH_2OCH_2CH_2OCH_3$ (**36**), NEt_3, DMF (c) BnBr, K_2CO_3, 100 °C, *34%* over 2 steps; (d) *N*-methylbenzyl amine, Cs_2CO_3, DMF, 100 °C, *85%*; (e) *4N* HCl, 1,4-dioxane, *93%*; (f) EtI, Cs_2CO_3, DMF, microwave, 130 °C, 30 min, *9%* (**40**); RBr, NaH, DMF, microwave, 130 °C, 30 min, *28-35%* (**41-43**).

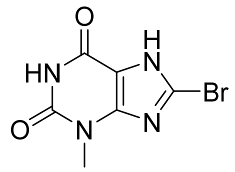

8-Bromo-3-methyl-3,7-dihydro-1*H*-purine-2,6-dione (35). Bromine (0.925 mL, 18.1 mmol, 3.0 eq) was added dropwise to a suspension of 3-methyl-3,7-dihydro-1*H*-purine-2,6-dione (**34**) (1.0 g, 6.0 mmol, 1.0 eq) in acetic acid (8.0 mL). The reaction was heated to 100 °C and stirred for 6 hours. Afterwards, the reaction was cooled to room temperature and washed with water. Residual water was removed under vacuum to afford 1.4 g (95%) of the title compound which was used further in the next step without purification.

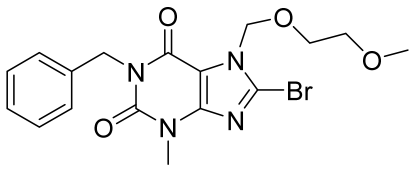

1-Benzyl-8-bromo-7-((2-methoxyethoxy)methyl)-3-methyl-3,7-dihydro-1*H*-purine-2,6-dione (37). Intermediate **35** (500 mg, 2.0 mmol, 1.1 eq) and triethylamine (0.56 mL, 4.0 mmol, 2.2 eq) were dissolved in DMF (3.0 mL), followed by the addition of MEM chloride (**36**) (206 µL, 1.8 mmol, 1.0 eq). The reaction was stirred overnight at room temperature. Upon the consumption of the starting material, BnBr (214 µL, 1.8 mmol, 1.0 eq) and K_2CO_3 (553 mg, 4.0 mmol, 2.2 eq) were added, and the reaction was stirred overnight at 100 °C. The reaction was cooled to room temperature, water was added, and the mixture was extracted with DCM (2x). The combined organics were washed with brine, dried over

Na_2SO_4, filtered, and concentrated *in vacuo*. Purification of the residue by flash chromatography on silica gel yielded 260 mg (34%) of the title compound. ^1H NMR (300 MHz, $CDCl_3$) δ 7.47 (m, 2H), 7.35 – 7.21 (m, 3H), 5.80 (s, 2H), 5.19 (s, 2H), 3.80 (m, 2H), 3.56 (s, 3H), 3.52 (m, 2H), 3.35 (s, 3H); LCMS R_T = 4.48 min; HRMS, calc'd for $C_{17}H_{20}BrN_4O_4^+$ [M+H], 423.0662; found 423.0669.

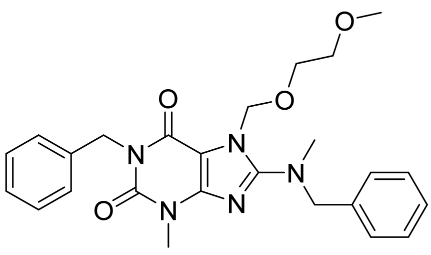

1-Benzyl-8-(benzyl(methyl)amino)-7-((2-methoxyethoxy)methyl)-3-methyl-3,7-dihydro-1*H*-purine-2,6-dione (38). Intermediate **37** (260 mg, 0.61 mmol, 1.0 eq), *N*-methylbenzyl amine (148 mg, 1.2 mmol, 2.0 eq), Cs_2CO_3 (397 mg, 1.2 mmol, 2.0 eq), and DMF (2.0 mL) were added to a microwave vial and heated in a microwave reactor at 130 °C for 30 minutes. The reaction was cooled to room temperature, water was added, and the mixture was extracted with ethyl acetate (2x). The combined organics were washed with brine, dried over Na_2SO_4, filtered, and concentrated *in vacuo*. Purification of the residue by flash chromatography on silica gel yielded 240 mg (85%) of the title compound. ^1H NMR (300 MHz, $CDCl_3$) δ 7.46 (m, 2H), 7.40 – 7.18 (m, 8H), 5.65 (s, 2H), 5.19 (s, 2H), 4.74 (s, 2H), 3.85 (m, 2H), 3.54 – 3.46 (m, 5H), 3.31 (m, 3H), 3.11 (s, 3H).

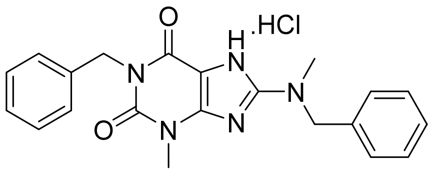

1-Benzyl-8-(benzyl(methyl)amino)-3-methyl-2,6-dioxo-2,3,6,7-tetrahydro-1*H*-purin-7-ium chloride (40). Intermediate **39** (240 mg, 0.52 mmol, 1.0 eq) was dissolved in 4*N* HCl in 1,4-dioxane (4.0 mL) and stirred at room temperature overnight. The solvent was removed *in vacuo* to afford 200 mg (93%) of the title compound, which was used further without purification.

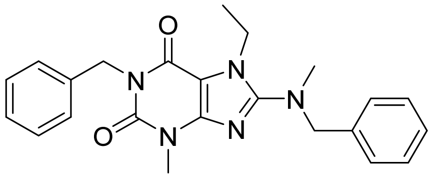

1-Benzyl-8-(benzyl(methyl)amino)-7-ethyl-3-methyl-3,7-dihydro-1H-purine-2,6-dione (41).

Intermediate **39** (35 mg, 0.085 mmol, 1.0 eq), iodoethane (26.5 mg, 0.17 mmol, 2.0 eq), Cs_2CO_3 (55.3 mg, 0.17 mmol, 2.0 eq), and DMF (0.5 mL) were added to a microwave vial and heated in a microwave reactor at 130 °C for 30 minutes. The reaction was cooled to room temperature, water was added, and the mixture was extracted with ethyl acetate (2x). The combined organics were washed with brine, dried over Na_2SO_4, filtered, and concentrated *in vacuo*. Purification of the residue by flash chromatography on silica gel yielded 3.0 mg (9%) of the title compound. ^1H NMR (300 MHz, $CDCl_3$) δ 7.49 (m, 2H),

7.42 – 7.17 (m, 8H), 5.19 (s, 2H), 4.43 (s, 2H), 4.22 (q, *J* = 7.10 Hz, 2H), 3.80 (s, 3H), 3.53 (s, 3H), 2.89 (s, 3H), 1.41 (t, *J* = 7.10 Hz, 3H). LCMS R_T = 5.50 min; HRMS, calc'd for $C_{23}H_{26}N_5O_2^+$ [M+H], 404.2081; found 404.2069.

The following compounds were prepared using a method analogous to that used for the conversion of intermediate **40** into compound **41**, using sodium hydride as base instead of cesium carbonate:

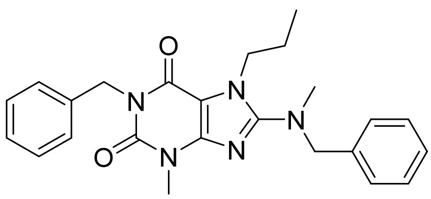

1-Benzyl-8-(benzyl(methyl)amino)-3-methyl-7-propyl-3,7-dihydro-1*H*-purine-2,6-dione (42). The title compound was prepared in 31% yield from intermediate **40** and 1-bromopropane. ^{1}H NMR (300 MHz, $CDCl_3$) δ 7.49 (m, 2H), 7.41 – 7.18 (m, 8H), 5.19 (s, 2H), 4.42 (s, 2H), 4.11 (m, 2H), 3.53 (s, 3H), 2.88 (s, 3H), 1.82 (m, 2H), 0.87 (t, *J* = 7.42 Hz, 3H); ^{13}C NMR (75 MHz, $CDCl_3$) δ 157.39, 154.31, 151.72, 148.10, 137.86, 136.94, 128.71, 128.34, 127.92, 127.72, 127.28, 104.58, 58.42, 47.18, 44.24, 39.61, 29.73, 23.69, 10.99 ppm. LCMS R_T = 5.72 min; HRMS, calc'd for $C_{24}H_{28}N_5O_2^+$ [M+H], 418.2238; found 418.2240.

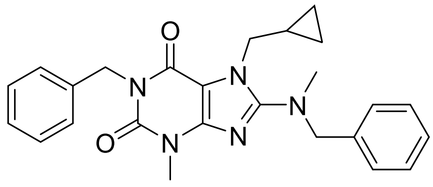

1-Benzyl-8-(benzyl(methyl)amino)-7-(cyclopropylmethyl)-3-methyl-3,7-dihydro-1*H*-purine-2,6-dione (43). The title compound was prepared in 28% yield from intermediate **40** and (bromomethyl)cyclopropane. ^{1}H NMR (300 MHz, $CDCl_3$) δ 7.48 (m, 2H), 7.40 – 7.18 (m, 8H), 5.20 (s, 2H), 4.43 (s, 2H), 4.09 (d, *J* = 7.08 Hz, 2H), 3.55 (s, 3H), 2.89 (s, 3H), 1.27 (m, 1H), 0.47 (m, 2H), 0.37 (m, 2H); ^{13}C NMR (75 MHz, $CDCl_3$) δ 157.53, 154.43, 151.70, 148.25, 137.86, 136.88, 128.74, 128.72, 128.33, 128.04, 127.74, 127.29, 104.59, 58.55, 49.77, 44.23, 39.75, 29.76, 11.50, 3.69 ppm. LCMS R_T = 5.74 min; HRMS, calc'd for $C_{25}H_{28}N_5O_2^+$ [M+H], 430.2238; found 430.2234.

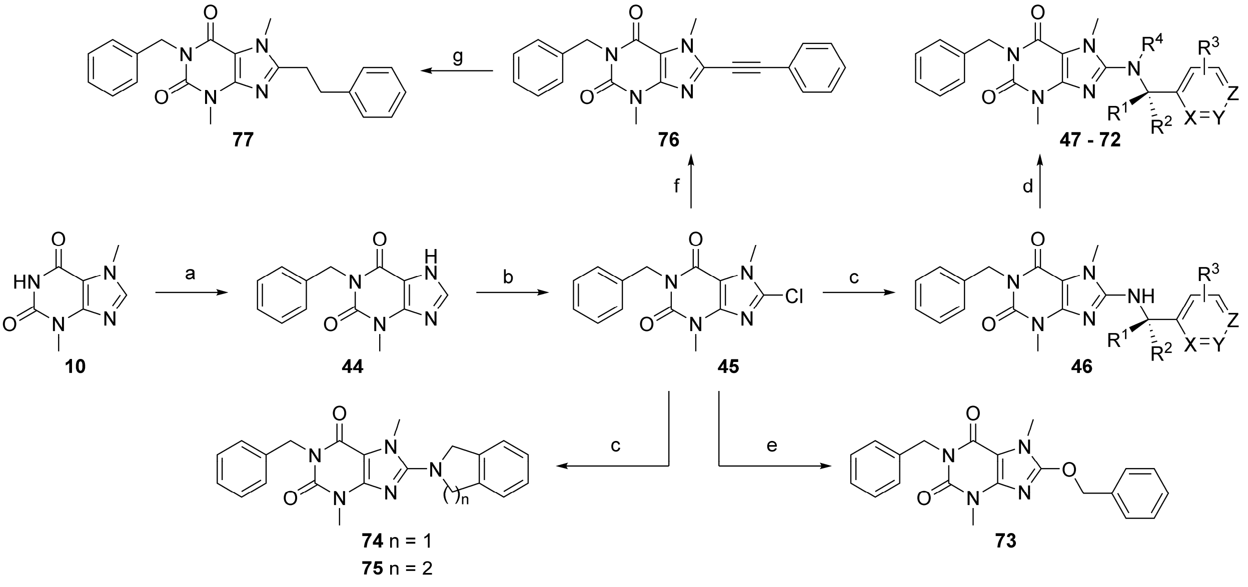

Scheme 3. Synthesis of western linker and ring analogs. *Reagents and Conditions:* (a) BnBr K_2CO_3, DMF, 90 °C, *95%*; (b) NCS, THF, 60 °C, *71%*; (c) 1° or 2° benzylamine, Cs_2CO_3, DMF, microwave, 130 °C, 30 min, *34%* (**74**), *32%* (**75**) (d) MeI, NaH, THF, *4 – 37%* over 2 steps (**47**-**66**); R^4X (X = Br or I), NaH, THF, 0 to 50 °C, *8-40%* (**67**-**72**); (e) $PhCH_2OH$, Cs_2CO_3, DMF, microwave, 130 °C, 30 min, *64%*; (f) PhC≡CH, $Pd(PPh_3)_4$, NEt_3, CuI, DMF, 100 °C, *47%*; (g) 10% Pd/C, MeOH, 10 bar, 60 °C, H-Cube®, *23%*.

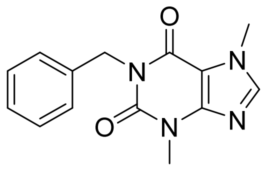

1-Benzyl-3,7-dimethyl-3,7-dihydro-1*H*-purine-2,6-dione (44). Theobromine (**10**) (1.58 g, 8.78 mmol, 1.5 eq) and K_2CO_3 (1.62 g, 11.7 mmol, 2.0 eq) were suspended in DMF (8.0 mL), followed by the addition of benzyl bromide (1.00 g, 5.85 mmol, 1.0 eq). The reaction was heated at 90 °C overnight. The reaction was cooled to room temperature, water was added, and the residue was filtered and rinsed with water. The product was dried at 40 °C to provide 1.50 g (95%) of the title compound as a white powder that was used further without purification. LCMS R_T = 3.98 min; HRMS, calc'd for $C_{14}H_{15}N_4O_2^+$ [M+H], 271.1190; found 271.1192.

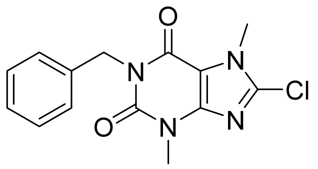

1-Benzyl-8-chloro-3,7-dimethyl-3,7-dihydro-1*H*-purine-2,6-dione (45). Intermediate **44** (1.50 g, 5.55, 1.0 eq) and NCS (1.48 g, 11.1 mmol, 2.0 eq) were suspended in THF (15 mL), and the reaction was heated at 60 °C overnight. THF was removed in vacuo, and the residue was filtered and washed with water. The product was dried at 40 °C to provide 1.31 g (78%) of the title compound as a beige solid that was used further without purification. ^{1}H NMR (300 MHz, $CDCl_3$) δ 7.47 (m, 2H), 7.40 – 7.18 (m, 3H), 5.18 (s, 2H), 3.95 (s, 3H), 3.53 (s, 3H). LCMS R_T = 4.64 min; HRMS, calc'd for $C_{14}H_{14}ClN_4O_2^+$ [M+H], 305.0800; found 305.0799.

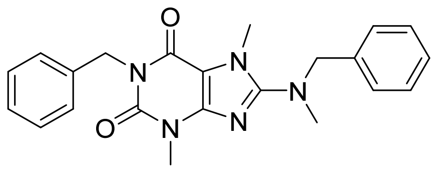

1-benzyl-8-(benzyl(methyl)amino)-3,7-dimethyl-3,7-dihydro-1*H*-purine-2,6-dione VU0607689 (8). Intermediate **45** (50 mg, 0.16 mmol, 1.0 eq), benzyl amine (34 mg, 0.32 mmol, 2.0 eq), Cs_2CO_3 (104 mg, 0.32 mmol, 2.0 eq), and DMF (0.5 mL) were added to a microwave vial and heated in a microwave reactor at 130 °C for 30 minutes. The reaction was cooled to room temperature, water was added, and the mixture was extracted with ethyl acetate (2x). The combined organics were washed with brine, dried over Na_2SO_4, filtered, and concentrated *in vacuo*. Purification of the residue by flash chromatography on silica gel yielded impure product that was dissolved in THF (2.0 mL), and cooled to 0 °C, followed by the addition of sodium hydride (15 mg, 0.64 mmol, 4.0 eq). The solution

was stirred for 30 minutes, followed by the addition of methyl iodide. The reaction temperature was brought to 50 °C and stirred at that temperature for 1 hour. The reaction was cooled to room temperature, water was added, and the mixture was extracted with DCM (2x). The combined organics were washed with brine, dried over Na_2SO_4, filtered, and concentrated *in vacuo*. Purification of the residue by flash chromatography on silica gel yielded 20 mg (32%) of the title compound.^{1}H NMR (300 MHz, $CDCl_3$) δ 7.49 (m, 2H), 7.41 – 7.18 (m, 8H), 5.19 (s, 2H), 4.45 (s, 2H), 3.80 (s, 3H), 3.52 (s, 3H), 2.90 (s, 3H); ^{13}C NMR (75 MHz, $CDCl_3$) δ 157.49, 154.61, 151.70, 147.83, 137.83, 136.79, 128.76, 128.74, 128.35, 127.86, 127.76, 127.32, 105.14, 57.48, 44.22, 38.93, 32.86, 29.70 ppm. LCMS R_T = 5.26 min; HRMS, calc'd for $C_{22}H_{24}N_5O_2^+$ [M+H], 390.1925; found 390.1932.

The following compounds **(47 – 64)** were prepared using a method analogous to that used for **8 (VU0607689)**.

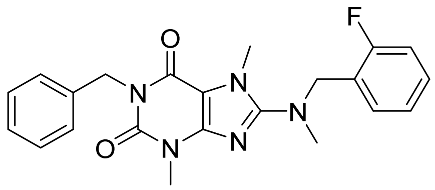

1-Benzyl-8-((2-fluorobenzyl)(methyl)amino)-3,7-dimethyl-3,7-dihydro-1*H*-purine-2,6-dione (47). The title compound was prepared in 11% yield from intermediate **45** and 2-fluorobenzylamine. ^{1}H NMR (300 MHz, $CDCl_3$) δ 7.49 (m, 2H), 7.41 – 7.19 (m, 5H), 7.18 – 7.01 (m, 2H), 5.18 (s, 2H), 4.50 (s, 2H), 3.79 (s, 3H), 3.52 (s, 3H), 2.92 (s, 3H); ^{13}C NMR (75

MHz, $CDCl_3$) δ 162.85, 159.58, 157.15, 154.63, 151.69, 147.74, 137.81, 130.18 (d, J(C,F) = 4.18 Hz), 129.60 (d, J(C,F) = 8.23 Hz), 128.54 (d, J(C,F) = 31.10 Hz), 127.32, 124.27 (d, J(C,F) = 3.66 Hz), 123.73 (d, J(C,F) = 14.32 Hz), 115.62 (d, J(C,F) = 21.57 Hz), 105.20, 51.09 (d, J(C,F) = 3.44 Hz), 44.22, 38.13, 32.76, 29.70 ppm. LCMS R_T = 5.37 min; HRMS, calc'd for $C_{22}H_{23}FN_5O_2^+$ [M+H], 408.1830; found 408.1831.

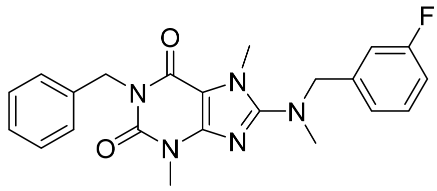

1-Benzyl-8-((3-fluorobenzyl)(methyl)amino)-3,7-dimethyl-3,7-dihydro-1*H*-purine-2,6-dione (48). The title compound was prepared in 15% yield from intermediate **45** and 3-fluorobenzylamine. ^{1}H NMR (300 MHz, $CDCl_3$) δ 7.49 (m, 2H), 7.39 – 7.18 (m, 4H), 7.16 – 6.94 (m, 3H), 5.19 (s, 2H), 4.45 (s, 2H), 3.81 (s, 3H), 3.52 (s, 3H), 2.90 (s, 3H); ^{13}C NMR (75 MHz, $CDCl_3$) δ 164.74, 161.48, 157.17, 154.64, 151.67, 147.70, 139.56 (d, J(C,F) = 6.79 Hz), 137.78, 130.28 (d, J(C,F) = 8.29 Hz), 128.54 (d, J(C,F) = 29.60 Hz), 127.33, 123.45 (d, J(C,F) = 2.81 Hz), 114.86 (d, J(C,F) = 3.09 Hz), 114.59 (d, J(C,F) = 2.29 Hz), 105.21, 57.01, 44.23, 39.15, 32.81, 29.70 ppm. LCMS R_T = 5.40 min; HRMS, calc'd for $C_{22}H_{23}FN_5O_2^+$ [M+H], 408.1830; found 408.1835.

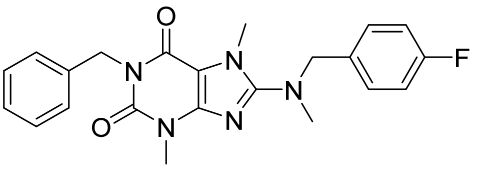

1-Benzyl-8-((4-fluorobenzyl)(methyl)amino)-3,7-dimethyl-3,7-dihydro-1*H*-purine-2,6-dione (49). The title compound was prepared in 17% yield from intermediate **45** and 4-fluorobenzylamine. ^{1}H NMR (300 MHz, $CDCl_3$) *δ* 7.49 (m, 2H), 7.36 – 7.19 (m, 5H), 7.05 (m, 2H), 5.18 (s, 2H), 4.40 (s, 2H), 3.80 (s, 3H), 3.52 (s, 3H), 2.87 (s, 3H); ^{13}C NMR (75 MHz, $CDCl_3$) δ 163.96, 160.70, 157.25, 154.63, 151.67, 147.73, 137.79, 132.50 (d, *J*(C,F) = 3.11 Hz), 129.68 (d, *J*(C,F) = 8.1 Hz), 128.55 (d, *J*(C,F) = 30.88 Hz), 127.34, 115.62 (d, *J*(C,F) = 21.49 Hz), 105.17, 56.79, 44.23, 38.93, 32.80, 29.70 ppm. LCMS R_T = 5.37 min; HRMS, calc'd for $C_{22}H_{23}FN_5O_2^+$ [M+H], 408.1830; found 408.1838.

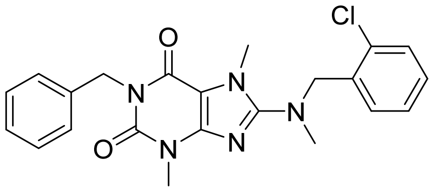

1-Benzyl-8-((2-chlorobenzyl)(methyl)amino)-3,7-dimethyl-3,7-dihydro-1*H*-purine-2,6-dione (50). The title compound was prepared in 4% yield from intermediate **45** and 2-chlorobenzylamine. ^{1}H NMR (300 MHz, $CDCl_3$) *δ* 7.48 (m, 2H), 7.45 – 7.36 (m, 2H), 7.34 – 7.19 (m, 5H), 5.18 (s, 2H), 4.56 (s, 2H), 3.74 (s, 3H), 3.52 (s, 3H), 2.96 (s, 3H); ^{13}C NMR (75 MHz, $CDCl_3$) δ 157.21, 154.60, 151.69, 147.77, 137.81, 134.45, 133.92, 129.90, 129.48, 129.01,

128.76, 128.34, 127.32, 126.99, 105.15, 55.08, 44.22, 39.51, 32.71, 29.71 ppm. LCMS R_T = 5.64 min; HRMS, calc'd for $C_{22}H_{23}ClN_5O_2^+$ [M+H], 424.1535; found 424.1540.

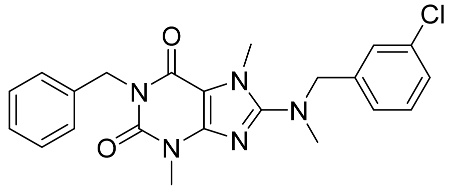

1-Benzyl-8-((3-chlorobenzyl)(methyl)amino)-3,7-dimethyl-3,7-dihydro-1*H*-purine-2,6-dione (51). The title compound was prepared in 12% yield from intermediate **45** and 3-chlorobenzylamine. ^{1}H NMR (300 MHz, $CDCl_3$) *δ* 7.49 (m, 2H), 7.39 – 7.16 (m, 7H), 5.19 (s, 2H), 4.43 (s, 2H), 3.81 (s, 3H), 3.52 (s, 3H), 2.30 (s, 3H); ^{13}C NMR (75 MHz, $CDCl_3$) δ 157.11, 154.64, 151.67, 147.68, 139.02, 137.78, 134.68, 130.02, 128.74, 128.35, 128.05, 127.97, 127.33, 126.06, 105.23, 56.97, 44.24, 39.18, 32.82, 29.71 ppm. LCMS R_T = 5.66 min; HRMS, calc'd for $C_{22}H_{23}ClN_5O_2^+$ [M+H], 424.1535; found 424.1539.

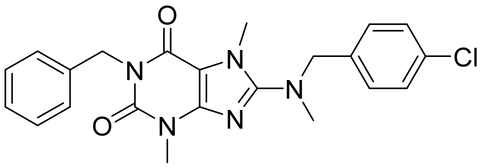

1-Benzyl-8-((4-chlorobenzyl)(methyl)amino)-3,7-dimethyl-3,7-dihydro-1*H*-purine-2,6-dione (52). The title compound was prepared in 19% yield from intermediate **45** and 4-chlorobenzylamine. ^{1}H NMR (300 MHz, $CDCl_3$) *δ* 7.49 (m, 2H), 7.38 – 7.18 (m, 7H), 5.18 (s, 2H), 4.41 (s, 2H), 3.80 (s, 3H), 3.51 (s, 3H), 2.88 (s, 3H); ^{13}C NMR (75 MHz, $CDCl_3$) δ

157.16, 154.63, 151.66, 147.70, 137.78, 135.34, 133.57, 129.33, 128.91, 128.76, 128.35, 127.34, 105.20, 56.85, 44.23, 39.08, 32.80, 29.70 ppm. LCMS R_T = 5.68 min; HRMS, calc'd for $C_{22}H_{23}ClN_5O_2^+$ [M+H], 424.1535; found 424.1540.

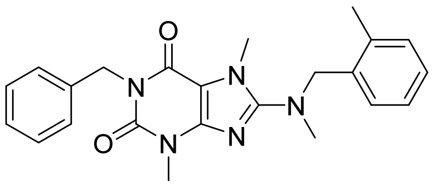

1-Benzyl-3,7-dimethyl-8-(methyl(2-methylbenzyl)amino)-3,7-dihydro-1*H*-purine-2,6-dione (53). The title compound was prepared in 19% yield from intermediate **45** and 2-methylbenzylamine. ^{1}H NMR (300 MHz, $CDCl_3$) *δ* 7.49 (m, 2H), 7.38 – 7.12 (m, 7H), 5.18 (s, 2H), 4.43 (s, 2H), 3.73 (s, 3H), 3.53 (s, 3H), 2.91 (s, 3H), 2.29 (s, 3H); ^{13}C NMR (75 MHz, $CDCl_3$) δ 157.60, 154.59, 151.70, 147.84, 137.83, 136.48, 134.75, 130.61, 128.75, 128.34, 128.08, 127.70, 127.32, 126.14, 105.12, 55.51, 44.21, 39.38, 32.72, 29.71, 19.12 ppm. LCMS R_T = 5.58 min; HRMS, calc'd for $C_{23}H_{26}N_5O_2^+$ [M+H], 404.2081; found 404.2089.

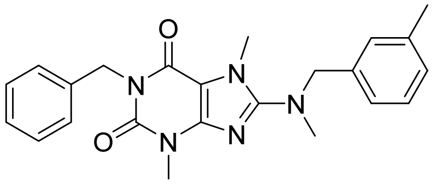

1-Benzyl-3,7-dimethyl-8-(methyl(3-methylbenzyl)amino)-3,7-dihydro-1*H*-purine-2,6-dione (54). The title compound was prepared in 11% yield from intermediate **45** and 3-methylbenzylamine. ^{1}H NMR (300 MHz, $CDCl_3$) *δ* 7.49 (m, 2H), 7.35 – 7.19 (m, 4H), 7.16

– 7.07 (m, 3H), 5.19 (s, 2H), 4.41 (s, 2H), 3.80 (s, 3H), 3.52 (s, 3H), 2.89 (s, 3H), 2.36 (s, 3H); ^{13}C NMR (75 MHz, $CDCl_3$) δ 157.55, 154.60, 151.71, 147.86, 138.45, 137.85, 136.72, 128.74, 128.65, 128.54, 128.50, 128.35, 127.31, 124.84, 105.12, 57.44, 44.21, 38.87, 32.90, 29.70, 21.48 ppm. LCMS R_T = 5.63 min; HRMS, calc'd for $C_{23}H_{26}N_5O_2^+$ [M+H], 404.2081; found 404.2084.

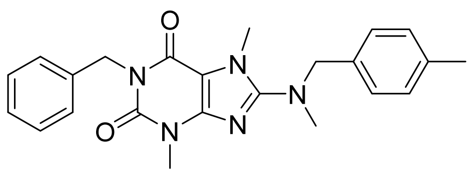

1-Benzyl-3,7-dimethyl-8-(methyl(4-methylbenzyl)amino)-3,7-dihydro-1*H*-purine-2,6-dione (55). The title compound was prepared in 33% yield from intermediate **45** and 4-methylbenzylamine. ^{1}H NMR (300 MHz, $CDCl_3$) *δ* 7.49 (m, 2H), 7.36 – 7.08 (m, 7H), 5.19 (s, 2H), 4.40 (s, 2H), 3.80 (s, 3H), 3.52 (s, 3H), 2.88 (s, 3H), 2.35 (s, 3H); ^{13}C NMR (75 MHz, $CDCl_3$) δ 157.54, 154.59, 151.71, 147.86, 137.85, 137.48, 133.68, 129.42, 128.74, 128.35, 127.85, 127.31, 105.11, 57.23, 44.21, 38.81, 32.87, 29.71, 21.15 ppm. LCMS R_T = 5.64 min; HRMS, calc'd for $C_{23}H_{26}N_5O_2^+$ [M+H], 404.2081; found 404.2084.

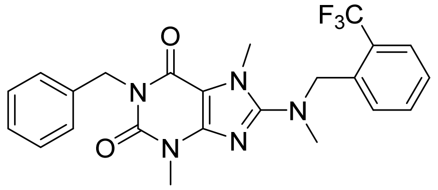

1-Benzyl-3,7-dimethyl-8-(methyl(2-(trifluoromethyl)benzyl)amino)-3,7-dihydro-1*H*-purine-2,6-dione (56). The title compound was prepared in 7% yield from intermediate

45 and 2-trifluorobenzylamine. ^{1}H NMR (300 MHz, $CDCl_3$) *δ* 7.70 (d, *J* = 7.74 Hz, 1H), 7.59 (m, 2H), 7.52 – 7.37 (m, 3H), 7.34 – 7.19 (m, 3H), 5.18 (s, 2H), 4.68 (s, 2H), 3.76 (s, 3H), 3.52 (s, 3H), 2.96 (s, 3H); ^{13}C NMR (75 MHz, $CDCl_3$) δ 157.17, 154.61, 151.68, 147.72, 135.76, 132.20, 128.85, 128.75, 128.35, 128.19, 127.68, 127.33, 126.44 (q, *J*(C, CF_3) = 5.86 Hz), 105.20, 53.90, 44.22, 39.61, 32.66, 29.70 ppm. LCMS R_T = 5.64 min; HRMS, calc'd for $C_{23}H_{23}F_3N_5O_2^+$ [M+H], 458.1798; found 458.1805.

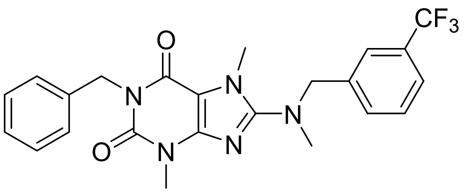

1-Benzyl-3,7-dimethyl-8-(methyl(3-(trifluoromethyl)benzyl)amino)-3,7-dihydro-1*H*-purine-2,6-dione (57). The title compound was prepared in 12% yield from intermediate **45** and 3-trifluorobenzylamine. ^{1}H NMR (300 MHz, $CDCl_3$) *δ* 7.64 (s, 1H), 7.61 – 7.43 (m, 5H), 7.35 – 7.18 (m, 3H), 5.19 (s, 2H), 4.50 (s, 2H), 3.82 (s, 3H), 3.52 (s, 3H), 2.91 (s, 3H); ^{13}C NMR (75 MHz, $CDCl_3$) δ 156.98, 154.65, 151.65, 147.61, 138.01, 137.75, 131.32, 129.22, 128.74, 128.35, 127.35, 124.89 (q, *J*(C, CF_3) = 3.82 Hz), 124.56 (q, *J*(C, CF_3) = 3.66 Hz), 122.20, 105.26, 57.08, 44.24, 39.36, 32.76, 29.68 ppm. LCMS R_T = 5.59 min; HRMS, calc'd for $C_{23}H_{23}F_3N_5O_2^+$ [M+H], 458.1798; found 458.1817.

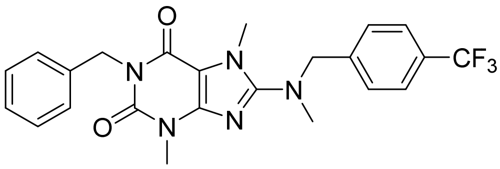

1-Benzyl-3,7-dimethyl-8-(methyl(4-(trifluoromethyl)benzyl)amino)-3,7-dihydro-1*H*-purine-2,6-dione (58). The title compound was prepared in 8% yield from intermediate **45** and 4-trifluorobenzylamine. ^{1}H NMR (300 MHz, $CDCl_3$) δ 7.63 (m, 2H), 7.48 (m, 4H), 7.35 – 7.19 (m, 3H), 5.18 (s, 2H), 4.51 (s, 2H), 3.82 (s, 3H), 3.51 (s, 3H), 2.91 (s, 3H); ^{13}C NMR (75 MHz, $CDCl_3$) δ 157.03, 154.65, 151.65, 147.65, 141.05,137.75, 130.28, 129.85, 128.76, 128.36, 128.17, (q, *J*(C, CF_3) = 3.82 Hz), 122.23, 105.26, 57.06, 44.24, 39.34, 32.79, 29.71 ppm. LCMS R_T = 5.62 min; HRMS, calc'd for $C_{23}H_{23}F_3N_5O_2^+$ [M+H], 458.1798; found 458.1807.

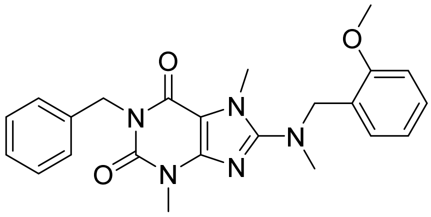

1-Benzyl-8-((2-methoxybenzyl)(methyl)amino)-3,7-dimethyl-3,7-dihydro-1*H*-purine-2,6-dione (59). The title compound was prepared in 23% yield from intermediate **45** and 2-methoxybenzylamine. ^{1}H NMR (300 MHz, $CDCl_3$) δ 7.48 (m, 2H), 7.36 – 7.18 (m, 5H), 7.02 – 6.83 (m, 2H), 5.19 (s, 2H), 4.45 (s, 2H), 3.77 (s, 3H), 3.76 (s, 3H), 3.52 (s, 3H), 2.94 (s, 3H); ^{13}C NMR (75 MHz, $CDCl_3$) δ 157.79, 157.75, 154.52, 151.74, 147.98, 137.91, 128.90, 128.78, 128.79, 128.33, 127.27, 124.76, 120.53, 110.37, 105.00, 55.26, 52.47, 44.18, 38.87, 32.84,

29.69 ppm. LCMS R_T = 5.28 min; HRMS, calc'd for $C_{23}H_{26}N_5O_3^+$ [M+H], 420.2030; found 420.2021.

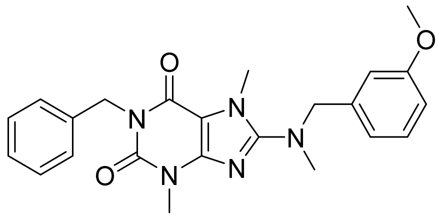

1-Benzyl-8-((3-methoxybenzyl)(methyl)amino)-3,7-dimethyl-3,7-dihydro-1*H*-purine-2,6-dione (60). The title compound was prepared in 20% yield from intermediate **45** and 3-methoxybenzylamine. ^{1}H NMR (300 MHz, $CDCl_3$) δ 7.49 (m, 2H), 7.36 – 7.17 (m, 4H), 6.96 – 6.80 (m, 3H), 5.19 (s, 2H), 4.42 (s, 2H), 3.80 (d, *J* = 2.06 Hz, 6H), 3.52 (s, 3H), 2.91 (s, 3H); ^{13}C NMR (75 MHz, $CDCl_3$) δ 159.96, 157.46, 154.60, 151.70, 147.82, 138.49, 137.83, 129.80, 128.72, 128.34, 127.31, 120.06, 113.69, 112.72, 105.13, 57.38, 55.25, 44.22, 38.96, 32.87, 29.70 ppm. LCMS R_T = 5.21 min; HRMS, calc'd for $C_{23}H_{26}N_5O_3^+$ [M+H], 420.2030; found 420.2029.

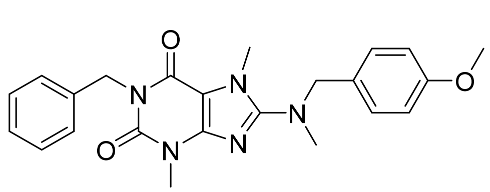

1-Benzyl-8-((4-methoxybenzyl)(methyl)amino)-3,7-dimethyl-3,7-dihydro-1*H*-purine-2,6-dione (61). The title compound was prepared in 25% yield from intermediate **45** and

4-methoxybenzylamine. ^{1}H NMR (300 MHz, $CDCl_3$) δ 7.49 (m, 2H), 7.35 – 7.17 (m, 5H), 6.89 (m, 2H), 5.19 (s, 2H), 4.37 (s, 2H), 3.80 (d, *J* = 4.31 Hz, 6H), 3.52 (s, 3H), 2.87 (s, 3H); ^{13}C NMR (75 MHz, $CDCl_3$) δ 159.20, 157.51, 154.60, 151.70, 147.84, 137.84, 129.27, 128.74, 128.67, 128.34, 127.31, 114.08, 105.11, 56.93, 55.31, 44.21, 38.69, 32.86, 29.71 ppm. LCMS R_T = 5.19 min; HRMS, calc'd for $C_{23}H_{26}N_5O_3^+$ [M+H], 420.2030; found 420.1999.

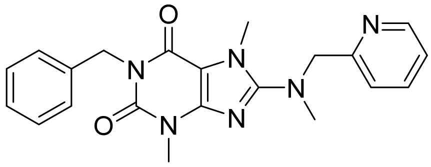

1-Benzyl-3,7-dimethyl-8-(methyl(pyridin-2-ylmethyl)amino)-3,7-dihydro-1*H*-purine-2,6-dione (62). The title compound was prepared in 37% yield from intermediate **45** and pyridin-2-ylmethanamine. ^{1}H NMR (300 MHz, $CDCl_3$) δ 8.60 (dq, *J* = 4.87, 0.85 Hz, 1H), 7.70 (dt, *J* = 7.69, 1.81 Hz, 1H), 7.48 (m, 2H), 7.41 – 7.17 (m, 5H), 5.18 (s, 2H), 4.60 (s, 2H), 3.83 (s, 3H), 3.51 (s, 3H), 3.03 (s, 3H); ^{13}C NMR (75 MHz, $CDCl_3$) δ 157.15, 156.99, 154.55, 151.69, 149.74, 147.83, 137.83, 136.88, 128.71, 128.33, 127.30, 122.65, 121.97, 105.14, 59.05, 44.20, 39.34, 32.98, 29.69 ppm. LCMS R_T = 3.66 min; HRMS, calc'd for $C_{21}H_{23}N_6O_2^+$ [M+H], 391.1877; found 391.1881.

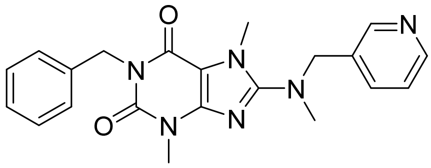

1-Benzyl-3,7-dimethyl-8-(methyl(pyridin-3-ylmethyl)amino)-3,7-dihydro-1*H*-purine-2,6-dione (63). The title compound was prepared in 26% yield from intermediate **45** and pyridin-3-ylmethanamine. ^{1}H NMR (300 MHz, $CDCl_3$) δ 8.59 (m, 2H), 7.69 (m, 1H), 7.48 (m, 2H), 7.35 – 7.18 (m, 4H), 5.18 (s, 2H), 4.47 (s, 2H), 3.81 (s, 3H), 3.51 (s, 3H), 2.90 (s, 3H); ^{13}C NMR (75 MHz, $CDCl_3$) δ 156.86, 154.65, 151.63, 149.79, 149.28, 147.57, 137.72, 135.88, 132.41, 128.73, 128.35, 127.35, 123.62, 105.28, 55.07, 44.24, 39.41, 32.73, 29.71 ppm. LCMS R_T = 3.41 min; HRMS, calc'd for $C_{21}H_{23}N_6O_2^+$ [M+H], 391.1877; found 391.1883.

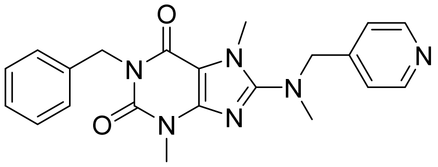

1-Benzyl-3,7-dimethyl-8-(methyl(pyridin-4-ylmethyl)amino)-3,7-dihydro-1*H*-purine-2,6-dione (64). The title compound was prepared in 6% yield from intermediate **45** and pyridin-4-ylmethanamine. ^{1}H NMR (300 MHz, $CDCl_3$) δ 8.60 (m, 2H), 7.49 (m, 2H), 7.36 – 7.18 (m, 5H), 5.19 (s, 2H), 4.47 (s, 2H), 3.82 (s, 3H), 3.50 (s, 3H), 2.94 (s, 3H); ^{13}C NMR (75 MHz, $CDCl_3$) δ 156.83, 154.66, 151.63, 150.24, 147.57, 146.16, 137.71, 128.75, 128.36, 127.37, 122.65, 105.31, 56.50, 44.25, 39.62, 32.77, 29.71 ppm. LCMS R_T = 3.36 min; HRMS, calc'd for $C_{21}H_{23}N_6O_2^+$ [M+H], 391.1877; found 391.1877.

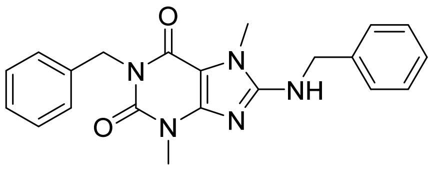

1-Benzyl-8-(benzylamino)-3,7-dimethyl-3,7-dihydro-1*H*-purine-2,6-dione (46a). Intermediate **45** (700 mg, 2.30 mmol, 1.0 eq), benzyl amine (493 mg, 4.60 mmol, 2.0 eq), Cs_2CO_3 (1.50 g, 4.60 mmol, 2.0 eq), and DMF (5.0 mL) were added to a microwave vial and heated in a microwave reactor at 130 °C for 30 minutes. The reaction was cooled to room temperature, water was added, and the mixture was extracted with ethyl acetate (2x). The combined organics were washed with brine, dried over Na_2SO_4, filtered, and concentrated *in vacuo*. Purification of the residue by flash chromatography on silica gel yielded 650 mg (38%) of the title compound. ^{1}H NMR (300 MHz, $CDCl_3$) δ 7.47 (m, 2H), 7.41 – 7.16 (m, 8H), 5.18 (s, 2H), 4.66 (d, *J* = Hz, 2H), 4.40 (m, 1H), 3.66 (s, 3H), 3.52 (s, 3H); ^{13}C NMR (75 MHz, $CDCl_3$) δ 154.12, 153.21, 151.69, 148.73, 138.13, 137.92, 128.87, 128.61, 128.34, 127.98, 127.26, 103.37, 47.43, 44.11, 29.72, 29.63 ppm. LCMS R_T = 4.87 min; HRMS, calc'd for $C_{21}H_{22}N_5O_2^+$ [M+H], 376.1768; found 376.1774.

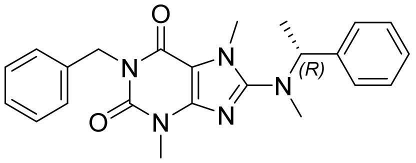

***(R)*-1-benzyl-3,7-dimethyl-8-(methyl(1-phenylethyl)amino)-3,7-dihydro-1*H*-purine-2,6-dione (65).** The title compound was prepared in 33% yield from intermediate **45** and (*R*)-1-phenylethylamine using a method analogous to that described for the conversion of intermediate **45** into compound **8**. ^{1}H NMR (300 MHz, $CDCl_3$) δ 7.50 (m, 2H), 7.44 – 7.19 (m, 8H), 5.19 (s, 2H), 4.90 (q, *J* = 6.84 Hz, 1H), 3.82 (s, 3H), 3.52 (s, 3H), 2.68 (s, 3H), 1.56 (d, *J* = 6.85 Hz, 3H); ^{13}C NMR (75 MHz, $CDCl_3$) δ 157.46, 154.66, 151.72, 147.90, 140.90, 137.85, 128.78, 128.69, 128.35, 127.59, 127.32, 127.23, 105.00, 59.56, 44.22, 34.31, 32.83, 29.74, 17.27 ppm. LCMS R_T = 5.47 min; HRMS, calc'd for $C_{23}H_{26}N_5O_2^+$ [M+H], 404.2081; found 404.2080.

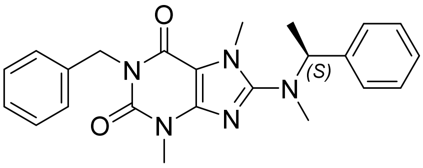

***(S)*-1-benzyl-3,7-dimethyl-8-(methyl(1-phenylethyl)amino)-3,7-dihydro-1H-purine-2,6-dione (66).** The title compound was prepared in 25% yield from intermediate **45** and (*S*)-1-phenylethylamine using a method analogous to that described for the conversion of intermediate **45** into compound **8**. ^{1}H NMR (300 MHz, $CDCl_3$) δ 7.50 (m, 2H), 7.44 – 7.19 (m, 8H), 5.19 (s, 2H), 4.90 (q, *J* = 6.84 Hz, 1H), 3.82 (s, 3H), 3.52 (s, 3H), 2.68 (s, 3H), 1.56 (d, *J* = 6.85 Hz, 3H); ^{13}C NMR (75 MHz, $CDCl_3$) δ 157.46, 154.66, 151.72, 147.90, 140.90, 137.85, 128.78, 128.69, 128.35, 127.59, 127.32, 127.23, 105.00, 59.56, 44.22, 34.31, 32.83, 29.74,

17.27 ppm. LCMS R_T = 5.47 min; HRMS, calc'd for $C_{23}H_{26}N_5O_2^+$ [M+H], 404.2081; found 404.2087.

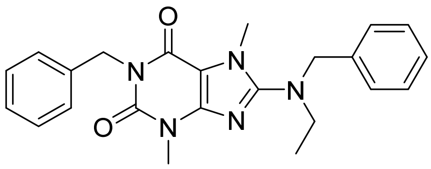

1-Benzyl-8-(benzyl(ethyl)amino)-3,7-dimethyl-3,7-dihydro-1H-purine-2,6-dione (67). Compound **46a** (50 mg, 0.13 mmol, 1.0 eq) was dissolved in THF (2.0 mL), and cooled to 0 °C, followed by the addition of NaH (12.5 mg, 0.52 mmol, 4.0 eq). The solution was stirred for 30 minutes, followed by the addition of iodoethane. The reaction temperature was brought to 50 °C and stirred for 1 hour. The reaction was cooled to room temperature, water was added, and the mixture was extracted with DCM (2x). The combined organics were washed with brine, dried over Na_2SO_4, filtered, and concentrated *in vacuo*. Purification of the residue by flash chromatography on silica gel yielded 21 mg (40%) of the title compound. ^{1}H NMR (300 MHz, $CDCl_3$) δ 7.49 (m, 2H), 7.39 – 7.18 (m, 8H), 5.18 (s, 2H), 4.54 (s, 2H), 3.76 (s, 3H), 3.51 (s, 3H), 3.27 (q, *J* = 7.11 Hz, 2H), 1.15 (t, *J* = 7.11 Hz, 3H); ^{13}C NMR (75 MHz, $CDCl_3$) δ 156.56, 154.62, 151.69, 147.78, 137.83, 137.44, 128.77, 128.62, 128.34, 127.95, 127.59, 127.32, 105.10, 54.71, 46.00, 44.21, 32.58, 29.71, 12.68 ppm. LCMS R_T = 5.51 min; HRMS, calc'd for $C_{23}H_{26}N_5O_2^+$ [M+H], 404.2081; found 404.2088.

Compounds **68 – 72** were prepared using a method analogous to that used for **compound 67**.

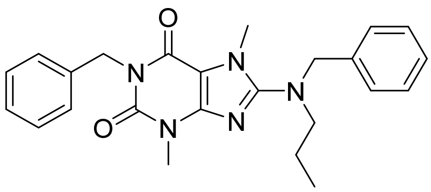

1-Benzyl-8-(benzyl(propyl)amino)-3,7-dimethyl-3,7-dihydro-1*H*-purine-2,6-dione (68). The title compound was prepared in 32% yield from compound **46a** and 1-bromopropane. ^{1}H NMR (300 MHz, $CDCl_3$) δ 7.49 (m, 2H), 7.38 – 7.17 (m, 8H), 5.18 (s, 2H), 4.46 (s, 2H), 3.77 (s, 3H), 3.51 (s, 3H), 3.17 (m, 2H), 1.58 (m, 2H), 0.86 (t, J = 7.40 Hz, 3H); ^{13}C NMR (75 MHz, $CDCl_3$) δ 156.72, 154.61, 151.69, 147.79, 137.84, 137.42, 128.79, 128.62, 128.34, 127.95, 127.60, 127.32, 105.00, 55.30, 53.18, 44.20, 32.61, 29.71, 20.77, 11.32 ppm. LCMS R_T = 5.76 min; HRMS, calc'd for $C_{24}H_{28}N_5O_2^+$ [M+H], 418.2238; found 418.2245.

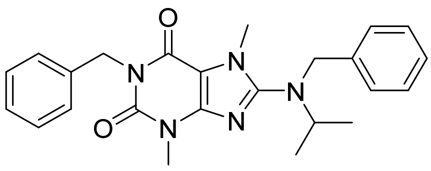

1-Benzyl-8-(benzyl(isopropyl)amino)-3,7-dimethyl-3,7-dihydro-1*H*-purine-2,6-dione (69). The title compound was prepared in 8% yield from compound **46a** and 2-bromopropane. ^{1}H NMR (300 MHz, $CDCl_3$) δ 7.47 (m, 2H), 7.33 – 7.14 (m, 8H), 5.14 (s, 2H), 4.35 (s, 2H), 3.67 (s, 3H), 3.60 (m, 1H), 3.49 (s, 3H), 1.29 (d, J = 6.65 Hz, 6H); ^{13}C NMR (75 MHz, $CDCl_3$) δ 155.93, 154.73, 151.64, 147.43, 138.80, 137.78, 128.83, 128.31, 128.06,

127.32, 127.15, 105.19, 54.09, 49.16, 44.19, 32.09, 29.68, 20.08 ppm. LCMS R_T = 5.66 min; HRMS, calc'd for $C_{24}H_{28}N_5O_2^+$ [M+H], 418.2238; found 418.2248.

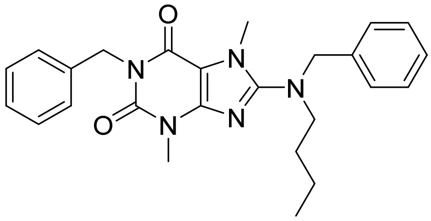

1-Benzyl-8-(benzyl(butyl)amino)-3,7-dimethyl-3,7-dihydro-1*H*-purine-2,6-dione (70). The title compound was prepared in 8% yield from compound **46a** and 1-bromobutane. ^{1}H NMR (300 MHz, $CDCl_3$) δ 7.49 (m, 2H), 7.39 – 7.18 (m, 8H), 5.18 (s, 2H), 4.45 (s, 2H), 3.77 (s, 3H), 3.51 (s, 3H), 3.20 (m, 2H), 1.54 (m, 2H), 1.27 (m, 2H), 0.88 (t, *J* = 7.31 Hz, 3H); ^{13}C NMR (75 MHz, $CDCl_3$) δ 156.73, 154.61, 151.69, 147.79, 137.84, 137.42, 128.80, 128.62, 128.34, 127.97, 127.60, 127.32, 105.02, 55.29, 51.19, 44.21, 32.62, 29.72, 29.60, 20.08, 13.86 ppm. LCMS R_T = 6.02 min; HRMS, calc'd for $C_{25}H_{30}N_5O_2^+$ [M+H], 432.2394; found 432.2408.

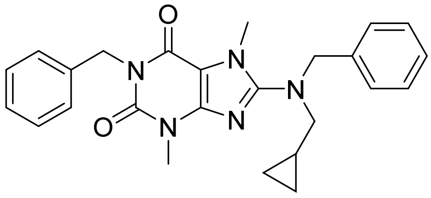

1-Benzyl-8-(benzyl(cyclopropylmethyl)amino)-3,7-dimethyl-3,7-dihydro-1*H*-purine-2,6-dione (71). The title compound was prepared in 29% yield from compound **46a** and (bromomethyl)cyclopropane. ^{1}H NMR (300 MHz, $CDCl_3$) δ 7.43 (m, 2H), 7.32 – 7.11 (m,

8H), 5.11 (s, 2H), 4.48 (s, 2H), 3.72 (s, 3H), 3.46 (s, 3H), 3.02 (d, *J* = 6.79 Hz, 2H), 0.94 (m, 1H), 0.42 (m, 2H), 0.01 (m, 2H); ^{13}C NMR (75 MHz, $CDCl_3$) δ 156.82, 154.68, 151.69, 147.73, 137.82, 137.49, 128.82, 128.59, 128.34, 127.91, 127.54, 127.33, 105.04, 56.32, 55.09, 44.23, 32.58, 29.74, 9.07, 3.67 ppm. LCMS R_T = 5.76 min; HRMS, calc'd for $C_{25}H_{28}N_5O_2^+$ [M+H], 430.2238; found 430.2251.

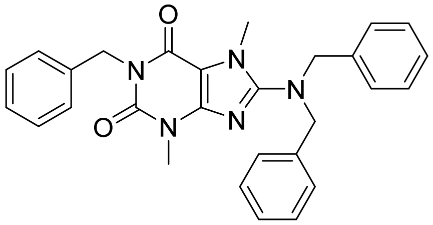

1-Benzyl-8-(dibenzylamino)-3,7-dimethyl-3,7-dihydro-1*H*-purine-2,6-dione (72). The title compound was prepared in 26% yield from compound **46a** and benzyl bromide. ^{1}H NMR (300 MHz, $CDCl_3$) *δ* 7.49 (m, 2H), 7.39 – 7.17 (m, 13H), 5.17 (s, 2H), 4.39 (s, 4H), 3.78 (s, 3H), 3.51 (s, 3H); ^{13}C NMR (75 MHz, $CDCl_3$) δ 156.35, 154.68, 151.66, 147.63, 137.78, 136.73, 128.86, 128.68, 128.35, 128.31, 127.79, 127.36, 105.10, 54.80, 44.24, 32.56, 29.75 ppm. LCMS R_T = 5.93 min; HRMS, calc'd for $C_{28}H_{28}N_5O_2^+$ [M+H], 466.2238; found 466.2246.

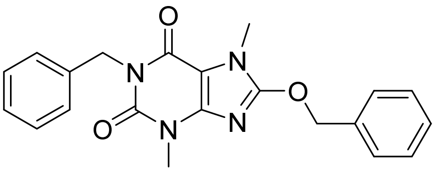

1-Benzyl-8-(benzyloxy)-3,7-dimethyl-3,7-dihydro-1*H*-purine-2,6-dione (73). The title compound was prepared in 64% yield from intermediate **45** and benzyl alcohol using a method analogous to that described for the conversion of intermediate **45** into compound **46a**. ^{1}H NMR (300 MHz, $CDCl_3$) δ 7.54 – 7.34 (m, 6H), 7.33 – 7.18 (m, 3H), 5.48 (s, 2H), 5.17 (s, 2H), 3.70 (s, 3H), 3.53 (s, 3H); ^{13}C NMR (75 MHz, $CDCl_3$) δ 155.64, 154.65, 151.59, 146.41, 137.67, 135.00, 128.91, 128.74, 128.73, 128.49, 128.36, 127.37, 103.63, 72.57, 44.23, 29.91, 29.80 ppm. LCMS R_T = 5.31 min; HRMS, calc'd for $C_{21}H_{21}N_4O_3^+$ [M+H], 377.1608; found 377.1600.

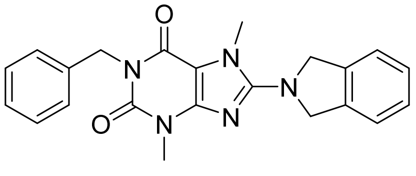

1-Benzyl-8-(isoindolin-2-yl)-3,7-dimethyl-3,7-dihydro-1*H*-purine-2,6-dione (74). The title compound was prepared in 34% yield from intermediate **45** and isoindoline hydrochloride using a method analogous to that described for the conversion of intermediate **45** into compound **46a**. ^{1}H NMR (300 MHz, $CDCl_3$) δ 7.49 (m, 2H), 7.38 – 7.18 (m, 7H), 5.19 (s, 2H), 5.02 (s, 4H), 4.01 (s, 3H), 3.53 (s, 3H). LCMS R_T = 5.35 min; HRMS, calc'd for $C_{22}H_{22}N_5O_2^+$ [M+H], 388.1768; found 388.1773.

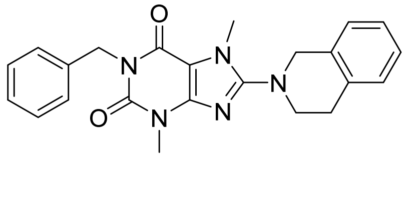

1-Benzyl-8-(3,4-dihydroisoquinolin-2(1H)-yl)-3,7-dimethyl-3,7-dihydro-1*H*-purine-2,6-dione (75). The title compound was prepared in 32% yield from intermediate **45** and 1,2,3,4-tetrahydroisoquinoline using a method analogous to that described for the conversion of intermediate **45** into compound **46a**. ^{1}H NMR (300 MHz, $CDCl_3$) δ 7.48 (m, 2H), 7.35 – 7.08 (m, 7H), 5.19 (s, 2H), 4.49 (s, 2H), 3.81 (s, 3H), 3.61 – 3.45 (m, 5H), 3.07 (t, *J* = 5.83 Hz, 2H); ^{13}C NMR (75 MHz, $CDCl_3$) δ 156.69, 154.74, 151.68, 147.81, 137.78, 133.65, 133.05, 129.00, 128.71, 128.36, 127.33, 126.80, 126.40, 126.34, 105.45, 51.21, 48.01, 44.25, 32.76, 29.76, 28.80 ppm. LCMS R_T = 5.54 min; HRMS, calc'd for $C_{23}H_{24}N_5O_2^+$ [M+H], 402.1925; found 402.1927.

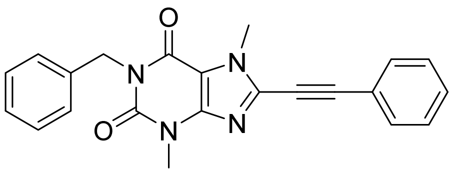

1-Benzyl-3,7-dimethyl-8-(phenylethynyl)-3,7-dihydro-1*H*-purine-2,6-dione (76). Intermediate **45** (150 mg, 0.49 mmol, 1.0 eq), ethynylbenzene (100 mg, 0.98 mmol, 2.0 eq), triethylamine (272 μL, 1.96 mmol, 4.0 eq), and DMF (2.0 mL) were charged in a flask, and the air was purged 3 times, followed by the addition of tetrakis(triphenylphosphine)palladium(0) ($Pd(PPh_3)_4$) (57 mg, 0.049 mmol, 0.10 eq) and copper iodide (CuI) (5 mg, 0.02 mmol, 0.05 eq). The reaction was heated at 100 °C overnight. The reaction was cooled to room temperature, water was added, and the mixture was extracted with DCM (2x). The combined organics were washed with brine,

dried over sodium sulfate (Na_2SO_4), filtered, and concentrated *in vacuo*. Purification of the residue by flash chromatography on silica gel yielded 85 mg (47%) of the title compound. ^{1}H NMR (300 MHz, $CDCl_3$) δ 7.62 (m, 2H), 7.54 – 7.36 (m, 5H), 7.36 – 7.21 (m, 3H), 5.21 (s, 2H), 4.09 (s, 3H), 3.59 (s, 3H); ^{13}C NMR (75 MHz, $CDCl_3$) δ 154.74, 151.46, 148.06, 137.20, 135.97, 132.14, 130.24, 128.88, 128.70, 128.45, 127.61, 120.43, 108.02, 97.32, 44.59, 33.27, 29.87 ppm. LCMS R_T = 5.42 min; HRMS, calc'd for $C_{22}H_{19}N_4O_2^+$ [M+H], 371.1503; found 371.1512.

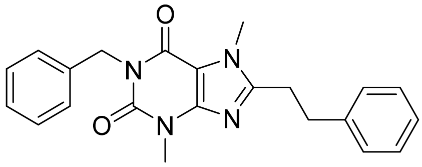

1-Benzyl-3,7-dimethyl-8-phenethyl-3,7-dihydro-1*H*-purine-2,6-dione (77). Compound **76** (26 mg, 0.07 mmol, 1.0 eq) was dissolved in methanol (5.0 mL) and subjected to H-cube® for hydrogenation under hydrogen pressure of 10 bar and 60 °C utilizing 10% Pd/C as a catalyst to afford 6 mg (23%) of the title compound. ^{1}H NMR (300 MHz, $CDCl_3$) δ 7.48 (m, 2H), 7.36 – 7.19 (m, 6H), 7.17 – 7.09 (m, 2H), 5.18 (s, 2H), 3.64 (s, 3H), 3.58 (s, 3H), 3.04 (m, 4H); ^{13}C NMR (75 MHz, $CDCl_3$) δ 155.11, 153.57, 151.65, 148.22, 140.01, 137.53, 128.80, 128.77, 128.39, 128.35, 127.45, 126.74, 107.27, 44.38, 34.01, 31.51, 29.78, 29.00 ppm. LCMS R_T = 5.06 min; HRMS, calc'd for $C_{22}H_{23}N_4O_2^+$ [M+H], 375.1816; found 375.1826.

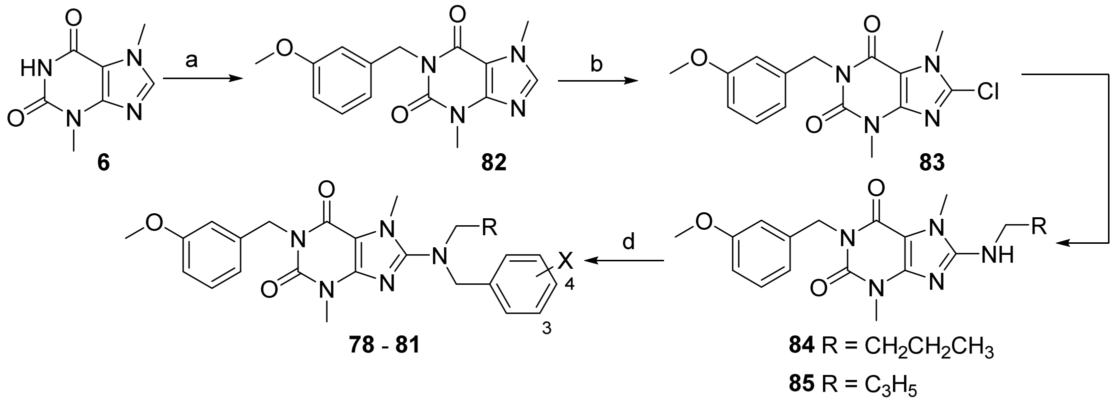

Reagents and conditions: (a) Cs_2CO_3, TBAI, DMF, 3-methoxybenzyl chloride, microwave, 140 °C, μW, *38%*; (b) NCS, THF, 60 °C, *83%*; (c) RNH_2, Cs_2CO_3, DMF, microwave, 130 °C, *52%* (**84**), *65%* (**85**); (d) $ArCH_2Br$, NaH, THF, *6-46%*.

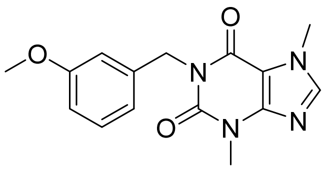

1-(3-methoxybenzyl)-3,7-dimethyl-3,7-dihydro-1*H*-purine-2,6-dione (82). Theobromine (1.56 g, 8.69 mmol, 1.1 eq) and C_2CO_3 (5.66 g, 17.4 mmol, 2.27 eq) were added to a microwave vial and suspended in DMF (7.0 mL), followed by the addition of 3-methoxybenzyl chloride (1.20 g, 7.66 mmol, 1.11 mL, 1.0 eq). The reaction was heated in a microwave reactor at 140 °C for 2 hours and cooled to room temperature. Water was added, and the aqueous layer was extracted with ethyl acetate and cold water. The organic layer was dried over Na_2SO_4, filtered, and concentrated *in vacuo*. The organic residue was purified using flash chromatography, which afforded 863 mg (*38%*) of the

title compound. ^{1}H NMR (300 MHz, $CDCl_3$) δ 7.51 (s, 1H), 7.25-7.20 (m, 1H), 7.07-7.02 (m, 2H), 6.81-6.77 (ddd, *J*=8.20, 2.60, 0.08 Hz, 1H), 5.18 (s, 2H), 3.99 (s, 3H), 3.79 (s, 3H), 3.58 (s, 3H); ^{13}C NMR (75 MHz, $CDCl_3$)LCMS R_T = 3.68 min; HRMS, calc'd for $C_{15}H_{17}N_4O_3^+$ [M+H], 301.1295; found 301.1298.

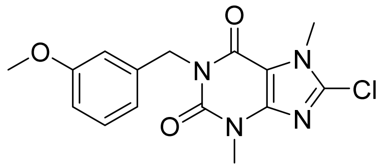

8-chloro-1-(3-methoxybenzyl)-3,7-dimethyl-3,7-dihydro-1H-purine-2,6-dione (83). Intermediate **82** (396 mg, 1.32, 1.0 eq) was suspended in THF (7 mL), followed by the portion-wise addition of NCS (191 mg, 1.45 mmol, 1.1 eq) at 0 °C, and the reaction was heated at 60 °C overnight. The reaction was allowed to come to room temperature. THF was removed *in vacuo*, and the residue was filtered and washed with water. The aqueous layer was extracted with ethyl acetate, and the combined organic layers were then dried over sodium sulfate, filtered, and concentrated *in vacuo.* Purification of the residue by flash chromatography provided 329 mg (83%) of the title compound. ^{1}H NMR (300 MHz, $CDCl_3$) δ 7.25-7.20 (m, 1H), 7.05-7.0 (m, 2H), 6.82-6.78 (ddd, *J*= 8.20, 2.80, 0.90 Hz, 1H), 5.16 (s, 2H) 3.95 (s, 3H), 3.79 (s, 3H), 3.54 (s, 3H). LCMS R_T = 4.04 min; HRMS, calc'd for $C_{15}H_{16}ClN_4O_3^+$ [M+H], 335.0905; found 335.0908.

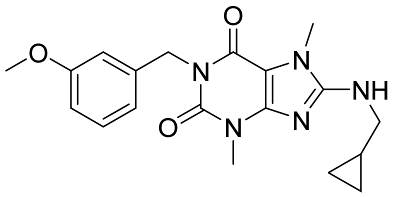

8-((cyclopropylmethyl)amino)-1-(3-methoxybenzyl)-3,7-dimethyl-3,7-dihydro-1*H*-purine-2,6-dione (85). Intermediate **83** (146 mg, 0.44 mmol, 1.0 eq), 1-cyclopropylmethanamine (0.08 mL, 1.75 mmol, 4.0 eq), and DMF (1.0 mL) were added to a microwave vial and heated in a microwave reactor at 130 °C for 1 h. The reaction was cooled to room temperature and diluted with toluene. The aqueous layer was extracted with ethyl acetate (2x). The combined organics were washed with brine, dried over Na_2SO_4, filtered, and concentrated *in vacuo*. Purification of the residue by flash chromatography (dichloromethane/ethyl acetate) on silica gel yielded 105 mg (65%) of product. ^1H NMR (300 MHz, $CDCl_3$) δ 7.23-7.18 (m, 1H), 7.04-7.0 (m, 2H), 6.77 (ddd, *J*= 8.1, 2.1, 0.9 Hz, 1H), 5.15 (s, 2H), 3.78 (s, 4H), 3.68 (s, 2H), 3.51 (m, 3H), 3.33 (t, *J*= 6.0 Hz, 1H), 2.97 (s, 2H), 1.25 (s, 2H), 1.11 (s, 1H), 0.58 (m, 1H), 0.31 (q, *J*=6.0 Hz, 1H).

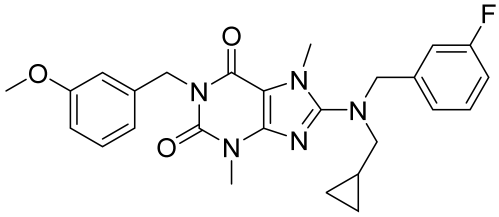

8-((Cyclopropylmethyl)(3-fluorobenzyl)amino)-1-(3-methoxybenzyl)-3,7-dimethyl-3,7-dihydro-1*H*-purine-2,6-dione (79). Intermediate **85** (0.105g, 0.28 mmol) was dissolved

at 0°C in THF (4.5 mL), followed by the addition of sodium hydride (0.05 g, 1.136mmol). The reaction was allowed to stir for 1 h. 3-fluorobenzyl bromide (0.109 mL, 0.82 mmol) was added dropwise to the solution. The reaction was allowed to stir at room temperature. THF was removed *in vacuo*, then the aqueous layer was extracted with DCM and brine (3x). The organic layer was dried over sodium sulfate, filtrate, and concentrated in vacuo. The product was purified using flash chromatography (hexane/ethyl acetate) to yield 16 mg (12%). ^{1}H NMR (300 MHz, $CDCl_3$) δ 7.21 (m, 2H), 7.02 (m, 5H), 6.78 (dd, *J*=9.0 Hz, 1H), 5.16 (s, 2H), 4.54 (s, 2H), 3.79 (d, *J*=1.11Hz, 6H), 3.51 (s, 3H), 3.07 (d, *J*=6.81, 2H), 0.97 (m, 1H), 0.50 (q, *J*=6.0 Hz, 2H), 0.09 (q, *J*=6.0, 3.0 Hz, 2H); ^{13}C NMR (75 MHz, $CDCl_3$) δ 164.64, 161.38, 159.56, 156.46, 154.65, 151.63, 147.61, 140.35 (d, *J(*C,F)=6.80 Hz), 139.30, 130.09 (d, *J(*C,F)=8.18 Hz), 129.34, 123.48 (d, *J(*C,F)=2.80 Hz), 120.94, 114.91, 114.61 (d, *J(*C,F)=1.90 Hz), 114.25 (d, *J(*C,F)=9.32 Hz), 112.73, 105.11, 56.72, 55.22, 54.57 (d, *J(*C,F)=1.58 Hz), 44.15, 32.55, 30.96, 29.74, 9.02, 3.68. LCMS R_T = min; HRMS, calc'd for $C_{26}H_{29}ClN_4O_3^+$ [M+H], 478.2249; found 478.2227.

The following compounds **78**, **80**, and **81** were prepared using a method analogous to that described for **79** above.

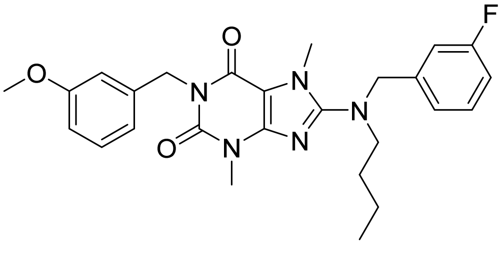

8-(Butyl(3-fluorobenzyl)amino)-1-(3-methoxybenzyl)-3,7-dimethyl-3,7-dihydro-1H-purine-2,6-dione (78). The title compound was prepared in 46% yield from intermediate **84** and 3-fluorobenzyl bromide. ^{1}H NMR (300 MHz, $CDCl_3$) δ 7.20-7.05 (m, 2H), 7.0-6.86 (m, 5H), 6.76 (ddd, *J*=8.2, 2.6, 0.8 Hz, 1H), 5.15 (s, 2H), 4.46 (s, 2H), 3.78 (d, *J*= 2.4 Hz, 6H), 3.50 (s, 3H), 3.20 (t, *J*=7.7Hz, 2H), 1.57-1.49 (m, 2H), 1.35-1.22 (m, 2H), 0.89 (t, *J*=7.7 Hz, 3H); ^{13}C NMR (75 MHz, $CDCl_3$) δ 164.63, 161.37, 159.56, 156.37, 154.58, 151.63, 147.66, 140.25 (d, *J*(C,F)=6.98 Hz), 139.33, 130.12 (d, *J*(C,F)=8.24 Hz), 129.34, 123.53 (d, *J*(C,F)=2.76 Hz), 120.92, 114.98, 114.68 (d, *J*(C,F)=1.63 Hz), 114.38, 114.18, 112.70, 105.09, 55.22, 54.75 (d, *J*(C,F)=1.65 Hz), 51.57, 44.13, 32.58, 29.65 (d, *J*(C,F)=10.35 Hz), 20.07, 13.84. LCMS R_T = min; HRMS, calc'd for $C_{26}H_{31}FN_5O_3^+$ [M+H], 480.2405; found 480.2389.

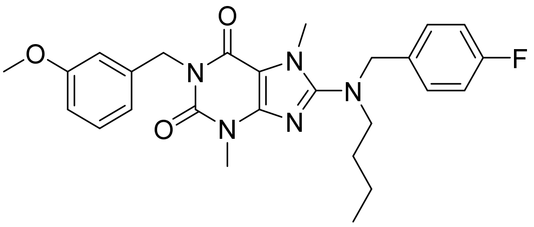

8-(Butyl(4-fluorobenzyl)amino)-1-(3-methoxybenzyl)-3,7-dimethyl-3,7-dihydro-1*H*-purine-2,6-dione (80). The title compound was prepared in 15% yield from intermediate **84** and 4-fluorobenzyl bromide. ^{1}H NMR (300 MHz, $CDCl_3$) δ 7.30-7.18 (m, 3H), 7.09-6.98 (m, 4H), 6.78 (ddd, *J*= 8.20, 2.60, 0.80 Hz, 1H), 5.15 (s, 2H), 4.41 (s, 2H), 3.77 (d, *J*= 5.20 Hz, 6H), 3.50 (s, 3H), 3.17 (t, *J*=7.60 Hz, 2H), 1.55-1.48 (m, 2H), 1.34-1.21 (m, 2H), 0.89 (t, *J*= 7.35 Hz, 3H); ^{13}C NMR (75 MHz, $CDCl_3$) δ 163.84, 160.58, 159.56, 156.46, 154.58, 151.63, 147.69,

139.34, 133.14 (d, *J*(C,F)=12.63 Hz), 129.73 (d, *J*(C,F)=7.93 Hz), 129.34, 120.94, 115.62, 115.34, 114. 21, 112.67, 105.04, 99.99, 55.22, 54.57, 51.33, 44.12, 32.54, 29.66 (d, *J*(C,F)= 9.77 Hz), 20.08, 13.85. HRMS, calc'd for $C_{26}H_{31}FN_5O_3^+$ [M+H], 480.2405; found 480.2411.

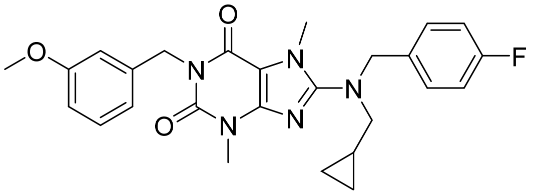

8-((cyclopropylmethyl)(4-fluorobenzyl)amino)-1-(3-methoxybenzyl)-3,7-dimethyl-3,7-dihydro-1*H*-purine-2,6-dione (81). The title compound was prepared in 6% yield from intermediate **85** and 4-fluorobenzyl bromide. ^{1}H NMR (300 MHz, $CDCl_3$) δ 7.30-7.18 (m, 3H), 7.03-6.98 (m, 4H), 6.78 (ddd, *J*= 8.20, 2.60, 0.80 Hz, 1H), 5.15 (s, 2H), 4.50 (s, 2H), 3.78 (d, *J*= 4.60 Hz, 6H), 3.51 (s, 3H), 3.05 (d, *J*=6.80 Hz, 2H), 1.0-0.94 (m, 1H), 0.05 (m, 2H), 0.07 (q, *J*= 5.30 Hz, 2H); ^{13}C NMR (75 MHz, $CDCl_3$) δ 157.58, 154.54, 149.66, 137.33, 131.23, 127.67 (d, *J*(C,F)=7.92 Hz), 127.36, 118.98, 113.62, 113.34, 112.25, 110.73, 54.60, 53.25, 52.47, 42.17, 30.52, 27.77. LCMS R_T = min; HRMS, calc'd for $C_{26}H_{29}ClN_4O_3^+$ [M+H], 478.2249; found 478.2227.
